# Detection of Pneumococcal DNA in Blood by Polymerase Chain Reaction for Diagnosing Pneumococcal Pneumonia in Young Children From Low- and Middle-Income Countries

**DOI:** 10.1093/cid/cix145

**Published:** 2017-05-29

**Authors:** Susan C. Morpeth, Maria Deloria Knoll, J. Anthony G. Scott, Daniel E. Park, Nora L. Watson, Henry C. Baggett, W. Abdullah Brooks, Daniel R. Feikin, Laura L. Hammitt, Stephen R. C. Howie, Karen L. Kotloff, Orin S. Levine, Shabir A. Madhi, Katherine L. O’Brien, Donald M. Thea, Peter V. Adrian, Dilruba Ahmed, Martin Antonio, Charatdao Bunthi, Andrea N. DeLuca, Amanda J. Driscoll, Louis Peter Githua, Melissa M. Higdon, Geoff Kahn, Angela Karani, Ruth A. Karron, Geoffrey Kwenda, Sirirat Makprasert, Razib Mazumder, David P. Moore, James Mwansa, Sammy Nyongesa, Christine Prosperi, Samba O. Sow, Boubou Tamboura, Toni Whistler, Scott L. Zeger, David R. Murdoch, for the PERCH Study Group, Katherine L. O’Brien, Orin S. Levine, Maria Deloria Knoll, Daniel R. Feikin, Andrea N. DeLuca, Amanda J. Driscoll, Nicholas Fancourt, Wei Fu, Laura L. Hammitt, Melissa M. Higdon, E. Wangeci Kagucia, Ruth A. Karron, Mengying Li, Daniel E. Park, Christine Prosperi, Zhenke Wu, Scott L. Zeger, Nora L. Watson, Jane Crawley, David R. Murdoch, W. Abdullah Brooks, Hubert P. Endtz, Khalequ Zaman, Doli Goswami, Lokman Hossain, Yasmin Jahan, Hasan Ashraf, Stephen R. C. Howie, Bernard E. Ebruke, Martin Antonio, Jessica McLellan, Eunice Machuka, Arifin Shamsul, Syed M.A. Zaman, Grant Mackenzie, J. Anthony G. Scott, Juliet O. Awori, Susan C. Morpeth, Alice Kamau, Sidi Kazungu, Micah Silaba Ominde, Karen L. Kotloff, Milagritos D. Tapia, Samba O. Sow, Mamadou Sylla, Boubou Tamboura, Uma Onwuchekwa, Nana Kourouma, Aliou Toure, Shabir A. Madhi, David P. Moore, Peter V. Adrian, Vicky L. Baillie, Locadiah Kuwanda, Azwifarwi Mudau, Michelle J. Groome, Nasreen Mahomed, Henry C. Baggett, Somsak Thamthitiwat, Susan A. Maloney, Charatdao Bunthi, Julia Rhodes, Pongpun Sawatwong, Pasakorn Akarasewi, Donald M. Thea, Lawrence Mwananyanda, James Chipeta, Phil Seidenberg, James Mwansa, Somwe wa Somwe, Geoffrey Kwenda, Trevor P. Anderson, Joanne Mitchell

**Affiliations:** 1 Kenya Medical Research Institute-Wellcome Trust Research Programme, Kilifi;; 2 Department of Infectious Disease Epidemiology, London School of Hygiene & Tropical Medicine, United Kingdom;; 3 Microbiology Laboratory, Middlemore Hospital, Counties Manukau District Health Board, Auckland, New Zealand;; 4 Department of International Health, International Vaccine Access Center, Johns Hopkins Bloomberg School of Public Health, Baltimore, Maryland,; 5 Milken Institute School of Public Health, Department of Epidemiology and Biostatistics, George Washington University,; 6 The Emmes Corporation, Rockville, Maryland;; 7 Global Disease Detection Center, Thailand Ministry of Public Health–US Centers for Disease Control and Prevention Collaboration, Nonthaburi;; 8 Division of Global Health Protection, Center for Global Health, Centers for Disease Control and Prevention, Atlanta, Georgia,; 9 Department of International Health, Johns Hopkins Bloomberg School of Public Health, Baltimore, Maryland;; 10 International Centre for Diarrhoeal Disease Research, Bangladesh, Dhaka and Matlab;; 11 Division of Viral Diseases, National Center for Immunizations and Respiratory Diseases, Centers for Disease Control and Prevention, Atlanta, Georgia;; 12 Department of Paediatrics, University of Auckland,; 13 Centre for International Health, University of Otago, Dunedin, New Zealand;; 14 Medical Research Council Unit, Basse, The Gambia;; 15 Division of Infectious Disease and Tropical Pediatrics, Department of Pediatrics, Center for Vaccine Development, Institute of Global Health, University of Maryland School of Medicine, Baltimore,; 16 Bill & Melinda Gates Foundation, Seattle, Washington;; 17 Medical Research Council: Respiratory and Meningeal Pathogens Research Unit,; 18 Department of Science and Technology/National Research Foundation: Vaccine Preventable Diseases Unit, University of the Witwatersrand, Johannesburg, South Africa;; 19 Center for Global Health and Development, Boston University School of Public Health, Massachusetts;; 20 Department of Pathogen Molecular Biology, London School of Hygiene & Tropical Medicine,; 21 Microbiology and Infection Unit, Warwick Medical School, University of Warwick, Coventry, United Kingdom; Departments of; 22 Epidemiology,; 23 Mental Health,; 24 International Health, Center for Immunization Research, Johns Hopkins Bloomberg School of Public Health, Baltimore, Maryland;; 25 Department of Biomedical Sciences, School of Health Sciences, University of Zambia,; 26 Zambia Center for Applied Health Research and Development, Lusaka;; 27 Department of Paediatrics & Child Health, Chris Hani Baragwanath Academic Hospital and University of the Witwatersrand, Johannesburg, South Africa;; 28 Department of Pathology and Microbiology, University Teaching Hospital, Lusaka, Zambia;; 29 Centre pour le Développement des Vaccins, Bamako, Mali;; 30 Department of Biostatistics, Johns Hopkins Bloomberg School of Public Health, Baltimore, Maryland;; 31 Department of Pathology, University of Otago, and; 32 Microbiology Unit, Canterbury Health Laboratories, Christchurch, New Zealand; 33 Johns Hopkins Bloomberg School of Public Health, Baltimore, Maryland; 34 Bill & Melinda Gates Foundation, Seattle, Washington; 35 Centers for Disease Control and Prevention, Atlanta, Georgia; 36 The Emmes Corporation, Rockville, Maryland; 37 Nuffield Department of Clinical Medicine, University of Oxford, United Kingdom; 38 University of Otago, Christchurch, New Zealand; 39 International Centre for Diarrhoeal Disease Research, Bangladesh, Dhaka and Matlab, Bangladesh; 40 Medical Research Council, Basse, The Gambia; 41 KEMRI–Wellcome Trust Research Programme, Kilifi, Kenya; 42 Division of Infectious Disease and Tropical Pediatrics, Department of Pediatrics, Center for Vaccine Development, Institute of Global Health, University of Maryland School of Medicine, Baltimore, Maryland and Centre pour le Développement des Vaccins (CVD-Mali), Bamako, Mali; 43 Respiratory and Meningeal Pathogens Research Unit, University of the Witwatersrand, Johannesburg, South Africa; 44 Thailand Ministry of Public Health–US CDC Collaboration, Nonthaburi, Thailand; 45 Boston University School of Public Health, Boston, Massachusetts, and University Teaching Hospital, Lusaka, Zambia; 46 Canterbury Health Laboratory, Christchurch, New Zealand

**Keywords:** pneumonia, pneumococcus, PCR, blood, diagnosis.

## Abstract

**Background.:**

We investigated the performance of polymerase chain reaction (PCR) on blood in the diagnosis of pneumococcal pneumonia among children from 7 low- and middle-income countries.

**Methods.:**

We tested blood by PCR for the pneumococcal autolysin gene in children aged 1–59 months in the Pneumonia Etiology Research for Child Health (PERCH) study. Children had World Health Organization–defined severe or very severe pneumonia or were age-frequency–matched community controls. Additionally, we tested blood from general pediatric admissions in Kilifi, Kenya, a PERCH site. The proportion PCR-positive was compared among cases with microbiologically confirmed pneumococcal pneumonia (MCPP), cases without a confirmed bacterial infection (nonconfirmed), cases confirmed for nonpneumococcal bacteria, and controls.

**Results.:**

In PERCH, 7.3% (n = 291/3995) of cases and 5.5% (n = 273/4987) of controls were blood pneumococcal PCR-positive (*P* < .001), compared with 64.3% (n = 36/56) of MCPP cases and 6.3% (n = 243/3832) of nonconfirmed cases (*P* < .001). Blood pneumococcal PCR positivity was higher in children from the 5 African countries (5.5%–11.5% among cases and 5.3%–10.2% among controls) than from the 2 Asian countries (1.3% and 1.0% among cases and 0.8% and 0.8% among controls). Among Kilifi general pediatric admissions, 3.9% (n = 274/6968) were PCR-positive, including 61.7% (n = 37/60) of those with positive blood cultures for pneumococcus.

**Discussion.:**

The utility of pneumococcal PCR on blood for diagnosing childhood pneumococcal pneumonia in the 7 low- and middle-income countries studied is limited by poor specificity and by poor sensitivity among MCPP cases.

Microbiological confirmation of childhood pneumococcal pneumonia is difficult. A minority of cases are detected by blood culture even under ideal conditions [1]; sputum is not spontaneously produced by young children and can be contaminated by organisms carried in the nasopharynx; detection of pneumococcus in the nasopharynx may represent asymptomatic carriage; diagnostic serology is insensitive in children, and paired samples are difficult to obtain [2]; and urinary antigen tests are not sufficiently specific for use in young children [3, 4].

The development of molecular tools to diagnose pneumococcal disease has been slow. Some earlier gene targets were found to be nonspecific to *Streptococcus pneumoniae* due to its close homology with related noninvasive species [5–9]. Consequently, pneumococcal polymerase chain reaction (PCR) assays now commonly target the autolysin gene (*lytA*), which is regarded as more specific [9]. However, clinical specificity of this assay has been difficult to measure, particularly among children, the population who most need an improved diagnostic test. Two studies found no PCR-positive blood samples using *lytA*-targeted assays among healthy children, suggesting 100% specificity [4, 10].

We investigated the performance of pneumococcal PCR on blood specimens for diagnosing pneumococcal pneumonia in children from low- and middle-income countries as part of the Pneumonia Etiology Research for Child Health (PERCH) study.

## METHODS

### PERCH Participants

PERCH enrolled children aged 1–59 months from August 2011 to January 2014 (24 months at each site) at 9 study sites across 7 countries: Dhaka and Matlab, Bangladesh; Basse, The Gambia; Kilifi, Kenya; Bamako, Mali; Soweto, South Africa; Nakhon Phanom and Sa Kaeo, Thailand; and Lusaka, Zambia. Cases were hospitalized with World Health Organization (WHO)–defined severe or very severe pneumonia, and controls without case-defining pneumonia were randomly selected from the community. Controls were frequency matched to cases within the following age groups: 1 to <6 months, 6 to <12 months, 12 to <24 months, and 24 to <60 months. Identification and selection of cases and controls have been described previously [11].

### Kilifi General Pediatric Admissions

The Kenya site also evaluated children aged ≤14 years not enrolled in the PERCH study who were admitted to the general medical pediatric service at Kilifi County Hospital (KCH) from December 2010 until December 2013. The definition of pneumonia severity was the same as that used in the PERCH study, except that lower chest wall indrawing was not a requirement for children aged >5 years. Supplementary Figure 1 describes the population of Kilifi general pediatric admissions studied; all such children were offered admission blood tests, including blood cultures.

### Laboratory and Radiological Methods

Blood for PCR was collected at admission from both PERCH cases and KCH general admissions cases, alongside blood for culture, and from PERCH controls at enrollment. Blood samples for PCR were collected into dedicated *ethylenediaminetetraacetic acid* tubes. Detailed methods for the processing of PERCH samples can be found elsewhere in this supplement [12]. For the additional KCH children, 200 μL of whole blood was extracted by the manual spin-column method using the QIAamp DNA blood mini kit within a week of sample collection. Extracted DNA was frozen at −80°C until it underwent pneumococcal PCR.

Whole blood was evaluated for the presence of *S. pneumoniae* nucleic acid using a quantitative real-time PCR assay for the *lytA* gene based on the US Centers for Disease Control and Prevention (CDC) method [9].

Blood cultures were performed as described previously at all PERCH sites [12].

Nasopharyngeal swabs for pneumococcal culture and nasopharyngeal/oropharyngeal (NP/OP) swabs for respiratory pathogen multiplex PCR were collected from all cases and controls in PERCH and from those KCH general admissions cases meeting the WHO definition of severe or very severe pneumonia. Quantitative real-time PCR for respiratory pathogens was performed on NP/OP specimens, as described elsewhere [12].

In PERCH, lung aspirates were collected at select sites (The Gambia, South Africa, Mali, and Bangladesh), and pleural fluid was collected from cases when clinically indicated at all sites.

Among KCH general admission patients, cerebrospinal fluid (CSF) was collected from children with clinical suspicion of meningitis and from neonates with WHO-defined probable severe bacterial illness and cultured for bacterial and fungal pathogens using standard microbiological methods.

All PERCH laboratory assays were performed in the individual site laboratories in each country, with standardized quality control procedures and regular external quality assurance [12].

Chest radiographs (CXRs) were obtained from PERCH cases at enrollment and classified as consolidation, other infiltrate, both consolidation and other infiltrate, normal, or uninterpretable by a trained panel of radiologists and pediatricians [13, 14]. Chest radiographs from non-PERCH KCH children were obtained from those with pneumonia; results were reported from those interpreted according to WHO methods [13]. More non-PERCH CXRs became available after March 2012 when a mobile CXR unit was procured.

### Definitions

Microbiologically confirmed pneumococcal pneumonia (MCPP) PERCH cases had pneumococcus detected by blood culture; culture, antigen detection, or PCR of pleural fluid; or culture or PCR of lung aspirate. Cases confirmed for a nonpneumococcal bacterial infection had a positive culture of blood, lung aspirate, or pleural fluid, or PCR of lung aspirate or pleural fluid, for a pathogenic bacterium other than pneumococcus. Nonconfirmed cases had no bacterial pathogen detected by blood culture; culture, antigen detection, or PCR of pleural fluid; or culture or PCR of lung aspirate.

Pneumococcal conjugate vaccine (PCV) vaccinated was defined as having received at least 1 dose of vaccine.

Receipt of antibiotics prior to specimen collection was defined by having either a positive serum bioassay [12] or documented administration of antibiotics on the day of admission at the referral or study hospital prior to blood culture collection.

Human immunodeficiency virus (HIV) infection was defined in PERCH as detectable viral load or presence of HIV antibodies by serology (for children aged >12 months). In Kilifi, HIV antibody/antigen rapid immunochromatographic test results as per the Kenyan national scheme were used.

High-density pneumococcal NP/OP PCR density was defined as >6.9 log_10_ copies/mL [15].

A PERCH control was considered to have a respiratory tract illness if cough or runny nose were reported. Respiratory tract illness was also considered present if the child had (1) ear discharge, wheeze, or difficulty breathing, and (2) either fever (temperature ≥38.0°C or reported fever in the past 48 hours) or sore throat. No control satisfied the WHO criteria for severe or very severe pneumonia.

### Analysis

The proportion of blood samples positive by pneumococcal PCR was studied by clinical and laboratory characteristics. PERCH analyses were conducted overall and stratified by site and by case and control status. Site-specific analyses were performed using the chi-square or Fisher’s exact test. PERCH analyses with all sites combined were performed using logistic regression adjusted for PERCH site. Characteristics associated with whole-blood pneumococcal PCR positivity were evaluated among case and control groups at sites with at least 1 blood pneumococcal PCR-positive in that group. Logistic regression models used the Firth modified likelihood approach to minimize bias due to small sample size and to accommodate zero-frequency cells [16].

As part of a quality control process, the proportion of pneumococcal PCR-positive samples was examined over time by date of sample collection, date of DNA extraction, and date of PCR test for each site.

All PERCH analyses were performed using SAS 9.4; data were from the July 2015 PERCH data freeze. Analyses of the additional data from Kilifi were performed using STATA 13.1.

## ETHICAL CONSIDERATIONS

The PERCH study protocol was approved by the institutional review board or ethical review committee at each of the study site institutions and at The Johns Hopkins Bloomberg School of Public Health. The analysis of additional participants at the Kilifi site was approved by the ethical review committee at the Kenya Medical Research Institute. Parents or guardians of all participants provided written informed consent.

## RESULTS

### PERCH Study

In PERCH, whole-blood PCR results were available from 94.4% (n = 3995/4232) cases and 93.7% (n = 4987/5325) controls; 7.3% of cases and 5.5% of controls were PCR-positive for pneumococcus in blood (*P* < .001). Sensitivity among pneumococcal blood culture–positive cases was 68.2% (n = 30/44) and among MCPP cases was 64.3% (n = 36/56). Positivity among nonconfirmed cases (6.3%) and CXR-positive nonconfirmed cases (7.3%) was lower than among MCPP cases (*P* < .001) and cases confirmed for a nonpneumococcal bacterium (n = 12/107: 11.2%; *P* = .07 and *P* = .02, respectively) (Table 1 and Supplementary Table 1).

**Table 1.** Characteristics Associated With Whole-Blood Pneumococcal Polymerase Chain Reaction Positivity in PERCH, by Case and Control Groups

	All MCPP Cases^a^			Nonconfirmed Cases^b^			Confirmed Nonpneumococcal Bacterial Cases^c^			All Controls		
	No.	No. (%) WB+	aOR^d^	No.	No. (%) WB+	aOR	No.	No. (%) WB+	aOR	No.	No. (%) WB+	aOR
Overall	56	36 (64.3)	...	3832	243 (6.3)	...	98	12 (12.2)	...	4987	273 (5.5)	...
PERCH site												
Kenya	4	3 (75.0)	...	556	25 (4.5)	...	6	3 (50.0)	...	751	48 (6.4)	...
The Gambia	16	6 (37.5)	...	570	51 (8.9)	...	16	4 (25.0)	...	608	47 (7.7)	...
Mali	24	19 (79.2)	...	619	56 (9.0)	...	26	2 (7.7)	...	715	38 (5.3)	...
Zambia	7	4 (57.1)	...	494	37 (7.5)	...	23	2 (8.7)	...	603	31 (5.1)	...
South Africa	5	4 (80.0)	...	885	66 (7.5)	...	27	1 (3.7)	...	963	98 (10.2)	...
Thailand	0	0 (0)	...	218	3 (1.4)	...	0	0 (0)	...	622	5 (0.8)	...
Bangladesh	0	0 (0)	...	490	5 (1.0)	...	0	0 (0)	...	725	6 (0.8)	...
Age	...	*P* = .92	...	...	*P* = .12	...	...	*P* = .44	...	...	*P* = .85	...
1–5 mo	11	7 (63.6)	...	1570	96 (6.1)	...	40	3 (7.5)	...	1534	88 (5.7)	...
6–11 mo	14	10 (71.4)	1.3	869	68 (7.8)	1.46	27	3 (11.1)	1.25	1195	72 (6.0)	1.14
12–23 mo	17	9 (52.9)	0.76	854	52 (6.1)	1.27	19	3 (15.8)	2.46	1218	64 (5.3)	1.11
24–59 mo	14	10 (71.4)	0.83	539	27 (5.0)	1.06	12	3 (25.0)	4.35	1040	49 (4.7)	1.01
Sex	...	*P* = .28	...	...	*P* = .36	...	...	*P* = .80	...	...	*P* = .09	...
Female	27	21 (77.8)	2.01	1614	98 (6.1)	0.88	58	8 (13.8)	1.18	2491	150 (6.0)	1.24
Male	29	15 (51.7)	...	2218	145 (6.5)	...	40	4 (10.0)	...	2495	123 (4.9)	...
HIV infected	...	*P* = .89	...	...	***P*** **= .03**	...	...	***P*** **= .02**	...	...	*P* = .66	...
Yes	13	10 (76.9)	1.14	211	24 (11.4)	1.7	13	3 (23.1)	10.1	208	19 (9.1)	1.12
No	36	23 (63.9)	...	3299	198 (6.0)	...	75	8 (10.7)	...	4224	224 (5.3)	...
PCV vaccinated^e^	...	*P* = .36	...	...	*P* = .63	...	...	*P* = .66	...	...	*P* = .07	...
Yes	37	22 (59.5)	0.44	1949	142 (7.3)	0.92	49	6 (12.2)	1.55	2447	196 (8.0)	1.51
No	11	9 (81.8)	...	574	48 (8.4)	...	20	1 (5.0)	...	473	24 (5.1)	...
Very severe pneumonia	...	*P* = .13	...	...	*P* = .23	...	...	*P* = .84	...	...	...	...
Yes	32	23 (71.9)	2.73	1213	90 (7.4)	1.19	47	6 (12.8)	0.88	0	0 (0)	...
No	24	13 (54.2)	...	2619	153 (5.8)	...	51	6 (11.8)	...	0	0 (0)	...
Prior antibiotic use^f^	...	*P* = .92	...	...	*P* = .681	...	...	*P* = .45	...	...	*P* = .25	...
Yes	14	9 (64.3)	1.08	1504	100 (6.6)	1.07	49	5 (10.2)	1.74	111	9 (8.1)	1.5
No	40	25 (62.5)	...	2166	132 (6.1)	...	47	6 (12.8)	...	4582	253 (5.5)	...
NP culture positive for pneumococcus	...	*P* = .52	...	...	***P*** **= .002**	...	...	*P* = .51	...	...	***P*** **= .01**	...
Yes	44	28 (63.6)	1.74	2000	149 (7.5)	1.55	40	5 (12.5)	0.62	3420	205 (6.0)	1.46
No	11	7 (63.6)	...	1788	91 (5.1)	...	55	6 (10.9)	...	1518	63 (4.2)	...
Pneumococcus colonized (culture or PCR positive)	...	...	...	...	***P*** **≤ .001**	...	...	*P* = .18	...	...	***P*** **= .02**	...
Yes	54	35 (64.8)	...	2882	214 (7.4)	2.33	68	11 (16.2)	6.42	4042	238 (5.9)	1.57
No	0	0 (0)	...	897	27 (3.0)	...	26	0 (0.0)	...	908	34 (3.7)	...
Pneumococcal NP/OP PCR density >6.9 log_10_ copies/mL	...	*P* = .10	...	...	***P*** **= .002**	...	...	*P* = .69	...	...	*P* = .32	...
Yes	36	26 (72.2)	3.62	457	48 (10.5)	1.69	27	3 (11.1)	0.74	392	27 (6.9)	1.24
No	18	9 (50.0)	...	3306	192 (5.8)	...	68	8 (11.8)	...	4490	243 (5.4)	...
NP/OP PCR positive for any virus	...	*P* = .56	...	...	*P* = .15	...	...	*P* = .63	...	...	*P* = .50	...
Yes	51	32 (62.7)	0.32	3353	207 (6.2)	0.75	79	8 (10.1)	0.67	3853	211 (5.5)	1.11
No	3	3 (100)	...	414	33 (8.0)	...	16	3 (18.8)	...	1034	59 (5.7)	...
Hypoxemia^g^	...	*P* = .13	...	...	*P* = .89	...	...	*P* = .48	...	...	--	...
Yes	23	19 (82.6)	2.9	1446	102 (7.1)	0.98	50	4 (8.0)	0.62	NA	NA	NA
No	33	17 (51.5)	...	2377	141 (5.9)	...	47	8 (17.0)	...	NA	NA	NA
Died in hospital	...	*P* = .30	...	...	*P* = .85	...	...	*P* = .38	...	...	...	...
Yes	13	10 (76.9)	2.26	240	19 (7.9)	1.05	31	5 (16.1)	1.76	NA	NA	NA
No	43	26 (60.5)	...	3590	224 (6.2)	...	67	7 (10.4)	...	NA	NA	NA
CXR+^h^	...	*P* = .11	...	...	*P* = .05	...	...	*P* = .39	...	...	...	...
Yes	38	21 (55.3)	0.07	1745	127 (7.3)	1.33	50	8 (16.0)	2.01	NA	NA	NA
No	6	6 (100)	...	1525	82 (5.4)	...	26	2 (7.7)	...	NA	NA	NA
Alveolar consolidation on CXR	...	*P* = .43	...	...	*P* ≤ .001	...	...	*P* = .81	...	...	...	...
Yes	31	18 (58.1)	0.55	853	83 (9.7)	1.81	35	5 (14.3)	1.18	NA	NA	NA
No	13	9 (69.2)	...	2417	126 (5.2)	...	41	5 (12.2)	...	NA	NA	NA
WBC >15/mm^3^	...	*P* = .27	...	...	*P* = .93	...	...	*P* = .02	...	...	...	...
Yes	27	15 (55.6)	0.49	1383	81 (5.9)	0.99	40	2 (5.0)	0.13	NA	NA	NA
No	27	19 (70.4)	...	2277	148 (6.5)	...	54	10 (18.5)	...	NA	NA	NA
CRP ≥40mg/L	...	*P* = .25	...	...	*P* ≤ .001	...	...	*P* = .58	...	...	...	...
Yes	40	26 (65.0)	2.96	873	87 (10.0)	1.87	58	5 (8.6)	0.63	NA	NA	NA
No	8	3 (37.5)	...	2456	119 (4.8)	...	25	2 (8.0)	...	NA	NA	NA

Characteristics associated with whole-blood pneumococcal polymerase chain reaction positivity were evaluated among case and control groups at sites with at least 1 blood pneumococcal PCR-positive in that group. *P* value for association of study group with whole blood–positivity in a logistic regression model adjusted for site: microbiologically confirmed pneumococcal pneumonia (MCPP) versus nonconfirmed: *P* < .001; MCPP versus all controls: *P* < .001; MCPP versus confirmed nonpneumococcal bacterial cases *P* < .001; nonconfirmed versus all controls: *P* = .63; nonpneumococcal bacterial case versus all controls: *P* = .04. Bold indicates *P* < .05.

Abbreviations: aOR, adjusted odds ratio; CRP, C-reactive protein; CXR, chest radiograph; HIV, human immunodeficiency virus; MCPP, microbiologically confirmed pneumococcal pneumonia; NA, not applicable; NP, nasopharyngeal; OP, oropharyngeal; PCR, polymerase chain reaction; PCV, pneumococcal conjugate vaccine; PERCH, Pneumonia Etiology Research for Child Health; WB, whole blood; WBC, white blood cells.

^a^MCPP was defined as isolation of pneumococcus from blood culture; culture or PCR of lung aspirate or pleural fluid; or BinaxNOW antigen detection on pleural fluid.

^b^Nonconfirmed cases were defined as cases without isolation of bacteria from culture of blood, lung aspirate, or pleural fluid, or PCR of lung aspirate or pleural fluid.

^c^Confirmed nonpneumococcal bacterial case was defined as a case with any nonpneumococcal bacterial pathogen detected by blood culture, by lung aspirate culture or PCR, or by pleural fluid culture or PCR.

^d^Adjusted for site.

^e^PCV vaccinated defined as at least 1 dose. Restricted to PCV-using sites (Kenya, The Gambia, Mali, and South Africa).

^f^Prior use of antibiotics defined as serum bioassay positive, antibiotic administration at the referral facility, or antibiotic administration prior to blood specimen collection at the study facility.

^g^Hypoxemia was defined as <92% on room air (<90% at elevation, Zambia and South Africa) or a requirement for supplemental oxygen if a room air reading was not available.

^h^CXR+ defined as radiographic evidence of pneumonia (consolidation and/or other infiltrates).

**Figure 1.**
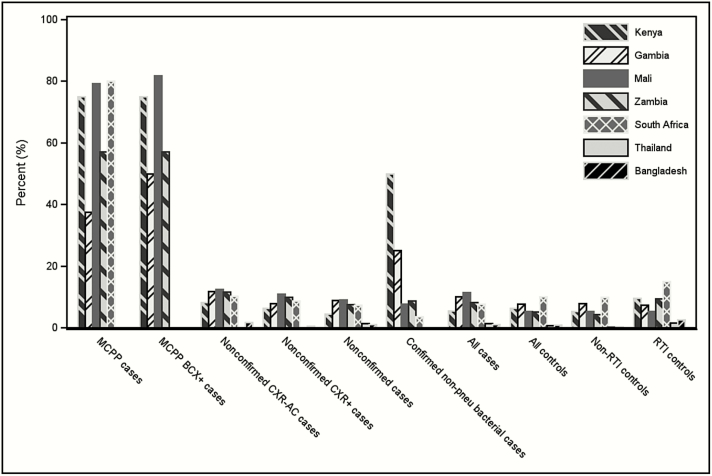
Proportion positive for pneumococcal whole-blood polymerase chain reaction (PCR), by PERCH site and case or control group. Microbiologically confirmed pneumococcal pneumonia (MCPP) cases were defined as isolation of pneumococcus from blood culture; culture or PCR of lung aspirate or pleural fluid; or BinaxNOW antigen detection on pleural fluid. Nonconfirmed cases were defined as cases without isolation of bacteria from culture of blood, lung aspirate, or pleural fluid, or PCR of lung aspirate or pleural fluid. Confirmed nonpneumococcal bacterial cases were defined as isolation of another bacteria from culture of blood, lung aspirate, or pleural fluid, or PCR of lung aspirate or pleural fluid. Chest X-ray–positive (CXR+) was defined as radiographic evidence of pneumonia (consolidation and/or other infiltrates). See Supplementary Table 1 with numeric results for each site and case/control group. *P* values from Fisher’s exact or chi-square test for difference in whole blood–positivity by site: MCPP: *P* = .08; nonconfirmed: *P* < .001; nonconfirmed CXR+: *P* < .001; confirmed nonpneumococcus bacterial case: *P* = .04; all controls: *P* < .001; controls with respiratory tract illness: *P* < .001; controls without acute respiratory illness: *P* < .001. Abbreviations: AC, alveolar consolidation; BCX+, blood culture positive; CXR, chest radiograph; MCPP, microbiologically confirmed pneumococcal pneumonia; PERCH, Pneumonia Etiology Research for Child Health; pneu, pneumococcus; RTI, controls with respiratory tract illness.

Positive blood pneumococcal PCR findings were observed at all sites among both cases and controls, including controls without respiratory tract illness; 2 sites (Kenya and South Africa) observed higher positivity in controls than in cases (Figure 1 and Supplementary Table 1). Blood pneumococcal PCR positivity was higher at the African sites (range, 5.5%–11.5% among cases and 5.3%–10.2% among controls) than at the 2 Asian sites (1.3% and 1.0% among cases and 0.8% and 0.8% among controls).

No association was found between pneumococcal PCR positivity in blood and age or sex. Blood pneumococcal PCR positivity tended to be higher overall among cases with HIV infection (Table 1 and Supplementary Tables 2–8). Among the 4 African sites that introduced PCV prior to PERCH, PCV use was associated with blood pneumococcal PCR positivity only in South Africa, where pneumococcal PCR positivity was lower among vaccinated versus unvaccinated cases (5.4% vs 9.5%; *P* = .04). No meaningful association with prior antibiotic use was observed.

Among both cases (7.4% vs 3.0%; *P* < .01) and controls (5.9% vs 3.7%; *P* = .02), blood pneumococcal PCR positivity was higher with versus without pneumococcus detected in the nasopharynx as measured by culture or PCR (Table 1). A quantitative nasopharyngeal PCR result above a threshold of 6.9 log_10_ copies/mL was significantly associated with blood pneumococcal PCR positivity among cases but not controls. The association between nasopharyngeal carriage and blood pneumococcal PCR positivity varied by site (Supplementary Tables 2–8).

Among nonconfirmed cases, higher proportions of blood pneumococcal PCR positivity were also observed for clinical findings typically associated with pneumococcal pneumonia: CXR-positive cases had higher positivity than CXR-negative cases (7.3% vs 5.4%; *P* = .05), with even higher positivity in the subset with consolidation on CXR (9.7%). This was also true in cases with C-reactive protein (CRP) ≥40 mg/L versus <40 mg/L (10.0% vs 4.8%; *P* < .001) (Table 1). Site-specific exceptions for association with CXR and CRP findings can be seen (Supplementary Tables 2–8). Analyses restricted to the CXR-positive nonconfirmed group were similar to analyses of the entire nonconfirmed case group (data not shown). The prevalence of pneumococcal PCR positivity in blood was also significantly higher among nonconfirmed cases with positive CXR (7.3%) or CRP ≥ 40 mg/L (10.0%) than among controls (5.5%; *P* < .01 for each comparison).

Low oxygen saturation, an elevated white blood cell count, and death in hospital were not significantly associated with blood pneumococcal PCR positivity overall or at any site.

### Kilifi General Pediatric Admissions

Of 6968 blood specimens collected from non-PERCH general pediatric admissions in Kilifi, 274 (3.9%) were whole-blood pneumococcal PCR-positive. Pneumococcal PCR in blood was positive among 61.7% (n = 37/60) of blood culture–positive cases and among 60.9% (n = 39/64) of blood culture– or CSF culture–positive cases. Among children least likely to have pneumococcal disease—those with a pathogen other than pneumococcus by culture of blood or CSF—4.7% (n = 7/148) were positive for pneumococcal PCR on blood, which was similar to the percentage observed in children with nonbacteremic pneumonia (4.2%) (Supplementary Table 9).

Neonates were the least likely to be pneumococcal PCR-positive in blood (2.1%; n = 29/1379), compared with children aged 29 days–59 months (4.3%; n = 177/4148) and children aged >59 months (4.7%; n = 68/1441; *P* = .003) (Table 2).

Table 2 and Table 3 describe characteristics associated with blood pneumococcal PCR positivity among children aged <5 years (Table 2) and aged 5–14 years (Table 3). Among children who did not have pneumococcal bacteremia and who did not have CSF collected, pneumonia status was associated with a positive pneumococcal blood PCR, with 4.2% of children aged 1–59 months with WHO-defined severe or very severe pneumonia positive compared with 2.7% without (*P* = .03). Clinical characteristics associated with blood pneumococcal PCR positivity among both children aged 1–59 months and children aged ≥60 months included HIV antibody positivity, nasopharyngeal pneumococcal carriage, and radiologically confirmed pneumonia. Children <60 months of age who died in hospital were more likely to have been blood pneumococcal PCR-positive than survivors (Table 2); this was not the case for older children (Table 3).

**Table 2.** Characteristics Associated With Whole-Blood Pneumococcal Polymerase Chain Reaction Positivity Among General Pediatric Admissions to Kilifi County Hospital, Aged <60 Months

			Children Aged 1–59 Months			
Characteristic	All Cases	All Cases Aged 0–28 days	Children With Pneumococcal Bacteremia^a^	Children Without Pneumococcal Bacteremia^a^		
				With Severe or Very Severe Pneumonia^b^	Without Severe or Very Severe Pneumonia ^b,c^	Confirmed for Another Pathogen^d^
	WB+ n/N (%)	WB+ n/N (%)	WB+ n/N (%)	WB+ n/N (%)	WB+ n/N (%)	WB+ n/N (%)
Overall	206/5527 (3.7)	29/1379 (2.1)	26/45 (57.8)	49/1173 (4.2)	47/1760 (2.7)	4/84 (4.8)
Age	***P* = .002**	…	*P* = .25	*P* = .82	*P* = .60	*P* > .99
0–28 d	29/1379 (2.1)	29/1379 (2.1)	NA	NA	NA	NA
29 d < 6 mo	31/781 (4.0)	NA	2/2 (100)	15/409 (3.7)	6/179 (3.4)	1/21 (4.8)
6–11 mo	38/790 (4.8)	NA	5/9 (55.6)	15/296 (5.1)	11/337 (3.3)	1/22 (4.6)
12–23 mo	40/1075 (3.7)	NA	9/12 (75.0)	11/257 (4.3)	10/517 (1.9)	1/24 (4.2)
24–59 mo	68/1502 (4.5)	NA	10/22 (45.5)	8/211 (3.8)	20/727 (2.8)	1/17 (5.9)
Pneumonia syndrome	***P* < .001**	*P* = .06	*P* = .03	*P* = .73	…	*P* = .59
Severe	50/901 (5.6)	9/212 (4.3)	11/13 (84.6)	25/627 (4.0)	NA	1/10 (10.0)
Very severe	53/1169 (4.5)	6/313 (1.9)	9/15 (60.0)	24/546 (4.4)	NA	1/22 (4.6)
Neither^c^	103/3455 (3.0)	14/853 (1.6)	6/17 (35.3)	NA	47/1760 (2.7)	2/52 (3.9)
HIV-antibody positive	***P* = .004**	*P* = .39	*P* = .68	*P* = .51	***P* = .02**	*P* = .46
Yes	19/269 (7.1)	2/60 (3.3)	7/13 (53.9)	1/59 (1.7)	6/94 (6.4)	0/12 (0.0)
No	168/4671 (3.6)	25/1149 (2.2)	17/28 (60.7)	44/1003 (4.4)	36/1473 (2.4)	4/57 (7.0)
NP culture or NP/OP PCR positive for pneumococcus	***P* = .003**	*P* = .12	*P* = .41	*P* = .09	…	…
Yes	40/771 (5.2)	3/45 (6.7)	10/16 (62.5)	21/528 (4.0)	NA	0/11 (0.0)
No	4/304 (1.3)	1/84 (1.2)	0/1 (0.0)	2/175 (1.1)	NA	0/4 (0.0)
NP/OP PCR positive for any virus^e^	*P* = .49	*P* = .32	*P* > .99	*P* > .99	…	…
Yes	29/782 (3.7)	3/56 (5.4)	7/13 (53.9)	14/546 (2.6)	NA	0/10 (0.0)
No	4/179 (2.2)	1/70 (1.4)	1/1 (100)	1/69 (1.5)	NA	0/1 (0.0)
CXR+^f^	***P* < .001**	…	…	***P* = .03**	…	…
Yes	9/46 (19.6)	NA	3/3 (100)	5/37 (13.5)	NA	1/3 (33.3)
No	11/238 (4.6)	NA	0/0 (0)	9/204 (4.4)	NA	0/5 (0.0)
Died in hospital	***P* = .03**	*P* = .95	*P* = .48	*P* = .48	*P* = .24	*P* = .63
Yes	24/415 (5.8)	4/196 (2.0)	7/10 (70.0)	5/89 (5.6)	3/64 (4.7)	1/18 (5.6)
No	182/5112 (3.6)	25/1183 (2.1)	19/35 (54.3)	44/1084 (4.1)	44/1969 (2.6)	3/66 (4.6)

*P* values obtained by chi-square or Fisher’s exact test for difference in whole blood pneumococcal PCR positivity by characteristic within each subgroup. Bold indicates *P* < .05.

Abbreviations: CXR, chest radiograph; HIV, human immunodeficiency virus; NA, not applicable; NP, nasopharyngeal; NP/OP, nasopharyngeal/oropharyngeal; PCR, polymerase chain reaction; WB, whole blood.

^a^Pneumococcus isolated on blood culture specimen.

^b^Children who did not have cerebrospinal fluid collected. No other pathogens were identified on blood culture.

^c^Not severe or very severe pneumonia syndrome includes children with any other medical cause of admission, including other respiratory illnesses. NP/OP swabs and CXRs were not obtained from these children.

^d^Confirmed for another pathogen by culture of blood or cerebrospinal fluid.

^e^ Respiratory viruses tested by multiplex PCR include respiratory syncytial virus A and B; parainfluenza viruses 1, 2, 3, and 4; coronaviruses OC43, NL63, 229E, and HKU; human metapneumovirus A and B; human bocavirus; influenza viruses A, B, and C; parecho/enterovirus; cytomegalovirus; and adenovirus.

^f^CXR results were included where children with WHO severe or very severe pneumonia had a digitalized CXR and standardized reporting according to WHO methods. A greater number of CXR results became available after March 2012 when a mobile CXR unit was procured.

**Table 3.** Characteristics Associated With Whole-Blood Pneumococcal Polymerase Chain Reaction Positivity Among General Pediatric Admissions to Kilifi County Hospital, Aged ≥60 Months

Characteristic	All Cases	Children With Pneumococcal Bacteremia^a^	Children Without Pneumococcal Bacteremia^a^		
			With Severe or Very Severe Pneumonia^b^	Without Severe or Very Severe Pneumonia^b,c^	Confirmed for Another Pathogen^d^
	WB+ n/N (%)	WB+ n/N (%)	WB+ n/N (%)	WB+ n/N (%)	WB+ n/N (%)
Overall	68/1441 (4.7)	10/13 (76.9)	14/184 (7.6)	33/850 (3.9)	2/24 (8.3)
Age	*P* = .80	*P* = .18	*P* = .39	*P* = .59	*P* = .37
5–7 y	37/800 (4.6)	5/5 (100)	7/88 (8.0)	20/443 (4.5)	1/12 (8.3)
8–10 y	19/426 (4.5)	4/5 (80.0)	3/64 (4.7)	8/264 (3.0)	0/9 (0.0)
11–14 y	12/215 (5.6)	1/3 (33.3)	4/32 (12.5)	5/143 (3.5)	1/3 (33.3)
Pneumonia syndrome	***P* = .02**	*P* > .99	*P* > .99	…	*P* > .99
Severe	6/71 (8.5)	1/1 (100)	5/65 (7.7)	NA	0/2 (0.0)
Very severe	15/188 (8.0)	5/6 (83.3)	9/119 (7.6)	NA	0/6 (0.0)
Neither^c^	47/1180 (4.0)	4/6 (66.7)	NA	33/850 (3.9)	2/16 (12.5)
HIV-antibody positive	***P* = .001**	*P* > .99	*P* = .06	*P* = .43	*P* = .40
Yes	13/116 (11.2)	2/2 (100)	6/38 (15.8)	3/51 (5.9)	1/5 (20.0)
No	49/1184 (4.1)	7/9 (77.8)	8/130 (6.2)	26/717 (3.6)	1/18 (5.6)
NP culture or NP/OP PCR positive for pneumococcus	*P* = .06	…	*P* = .42	…	…
Yes	11/115 (9.6)	5/5 (100)	6/74 (8.1)	NA	0/4 (0.0)
No	1/60 (1.7)	0/0 (0)	1/39 (2.6)	NA	0/2 (0.0)
NP/OP PCR positive for any virus^e^	*P* = .53	…	*P* > .99	…	…
Yes	5/94 (5.3)	1/1 (100)	4/61 (6.6)	NA	0/3 (0.0)
No	6/69 (8.7)	3/3 (100)	3/44 (6.8)	NA	0/2 (0.0)
CXR+^f^	***P* = .02**	…	…	…	…
Yes	3/14 (21.4)	3/3 (100)	0/9 (0.0)	NA	0/1 (0.0)
No	0/38 (0.0)	0/0 (0)	0/22 (0.0)	NA	0/2 (0.0)
Died in hospital	*P* > .99	*P* > .99	*P* = .69	*P* = .64	*P* > .99
Yes	3/77 (3.9)	1/1 (100)	2/23 (8.7)	0/34 (0.0)	0/4 (0.0)
No	65/1364 (4.8)	9/12 (75.0)	12/161 (7.5)	33/816 (4.0)	2/20 (10.0)

*P* values obtained by chi-square or Fisher’s exact test for difference in whole blood pneumococcal PCR positivity by characteristic within each subgroup. Bold indicates *P* < .05.

Abbreviations: CXR, chest radiograph; HIV, human immunodeficiency virus; NA, not applicable; NP, nasopharyngeal; NP/OP, nasopharyngeal/oropharyngeal; PCR, polymerase chain reaction; WB, whole blood.

^a^Pneumococcus isolated on blood culture specimen.

^b^Children who did not have cerebrospinal fluid collected. No other pathogens were identified on blood culture.

^c^Children without severe or very severe pneumonia syndrome had any other medical cause of admission, including other respiratory illnesses. NP/OP swabs and CXRs were not obtained from these children.

^d^Confirmed for another pathogen by culture of blood or cerebrospinal fluid.

^e^Respiratory viruses tested by multiplex PCR include respiratory syncytial virus A and B; parainfluenza viruses 1, 2, 3, and 4; coronaviruses OC43, NL63, 229E, and HKU; human metapneumovirus A and B; human bocavirus; influenza viruses A, B, and C; parecho/enterovirus; cytomegalovirus; and adenovirus.

^f^CXR results were included where children with WHO severe or very severe pneumonia had a digitalized CXR and standardized reporting according to WHO methods. A greater number of CXR results became available after March 2012 when a mobile CXR unit was procured.

### Assay Quality Control

Whole-blood pneumococcal PCR positivity was evaluated at each PERCH site over time, by date of sample collection, date of DNA extraction, and date of PCR testing, and no obvious trends in positivity were detected (Supplementary Figure 2–4).

## DISCUSSION

Pneumococcal PCR on blood was positive among 1%–10% of community controls in 7 African and Asian countries and is therefore not 100% specific for the diagnosis of pneumococcal disease as has been previously reported [4, 10]. The utility of pneumococcal PCR on blood in diagnosing childhood pneumococcal pneumonia is further limited by the fact that positivity among controls (5.5%) was similar to that in pneumonia cases not confirmed for any bacterial pathogen (6.3%), positivity among cases confirmed for nonpneumococcal bacteria (11.2%) was greater than that among nonconfirmed cases, and sensitivity among MCPP cases was low (64.3%).

There was a clear difference in the detection of pneumococcus in blood by PCR between the African and the Asian PERCH sites, with only 1.1% of nonconfirmed cases being pneumococcal PCR-positive at the Asian sites, compared with 7.5% at the African sites, and no MCPP cases in the Asian PERCH sites, despite common nasopharyngeal pneumococcal carriage among Thailand (57%) and Bangladesh (72%) cases. The reason for these differences between African and Asian sites is unclear, although it could be due to differences in transmission dynamics, host susceptibility, or prehospital antibiotic use. Other studies of hospitalized respiratory illness in Mali [17] and South Africa [18] have reported similar positivity for pneumococcal PCR on blood; 13.6% in Mali and 4%–6% among children aged <5 years in the South African study.

Findings from the extra sample set from Kilifi were consistent with those of the PERCH study in similarly aged children, providing support for the PERCH results. Furthermore these data showed analogous findings in children aged 5–14 years and also provided results in neonates, a population not often studied.

In contrast with our findings, previous studies in Italy and South Africa observed 100% specificity of the whole-blood *lytA* PCR assay despite half the children being colonized with pneumococcus, but these were much smaller studies (n = 147 and n = 100) and the study populations had important differences from PERCH [4, 10]. Controls in those studies were not a random selection from the community but were attending hospital for allergies or celiac disease [4] or had previously been vaccinated with PCV-9 in a clinical trial [10], were older (mean age of approximately 5 years), had lower prevalence (50%) [4, 10] and density [4] of nasopharyngeal pneumococcal carriage, and possibly had higher socioeconomic status. The South African study also used serum rather than whole blood [10]. In Slovenia, pneumococcal PCR was positive from plasma in 2 of 29 (6.9%) children with a nonpneumonia acute febrile illness for which a nonpneumococcal cause was identified [19]. Both of the children who tested positive were colonized with pneumococcus.

A review of older studies using a variety of gene targets to diagnose pneumococcal bacteremia from blood samples carried out during the period 1993–2009 describes sensitivity of 57%–66% and specificity of 88%–99% [20]. The South African respiratory illness study also reported positivity of 61% among blood culture–positive cases [18]. Poor sensitivity has been thought to be due to low specimen volume used by nucleic acid amplification tests, PCR inhibitors, and pneumococcal autolysis and DNA degradation from suboptimal storage conditions [8, 9, 21]. *lytA* has been shown to be a specific gene target for identifying pneumococcus for PCR assays [9, 21–24], and although the autolysin gene has been found in some *Streptococcus pseudopneumoniae* [25] and *Streptococcus mitis* genomes [7], it can be differentiated from the *lytA* gene in *S. pneumoniae* [22, 25]. The pneumococcal PCR method we used was published by the CDC in 2007 [9] and is in widespread use globally, so it is possible that other investigators have also used the assay in control groups and not published the results, perhaps assuming that there was laboratory error. However, the PERCH study used 7 different laboratories and found positive controls at every site.

We carefully examined the performance of the PCR assay on whole blood. We included all positive amplification curves up to a cycle threshold value of 40 cycles. The lower limit of reliable detection was 100–500 copies/mL of whole blood. Because the median *lytA* concentration in blood was on the lower end of this range, at 180 copies/mL among controls and 280 copies/mL among nonconfirmed cases [26], we expect that there would have been more positives if the assay were more sensitive.

The PERCH study invested in laboratory quality assurance, with implementation of a standard operating procedure aimed at reducing the potential for intralaboratory contamination and an external quality assurance program. Positive samples from control subjects were a feature of all 7 laboratories testing samples in the PERCH project, and it seems very unlikely that false positives due to intralaboratory contamination could explain all of these data. Dagan and colleagues suggested that pneumococcal DNA may be detectable in the bloodstream of healthy individuals who do not develop clinical disease from pneumococci which directly invaded the blood from the nasopharynx or which entered the bloodstream phagocytosed by lymphoid cells [5]. Although others have suggested that the positives among healthy controls in that study may have been due to the nonspecificity of the *ply* target gene used, our findings confirm positivity in healthy individuals with the more specific *lytA* target gene.

If we suppose that positive pneumococcal PCR results among control groups are due to the real presence of pneumococcal DNA in the bloodstream, then we must question our understanding of the pathophysiology of pneumococcal disease. Blood pneumococcal PCR positivity among controls was higher among those with nasopharyngeal carriage (5.9% vs 3.7%; *P* = .02): perhaps pneumococci invade through the mucosa of the nasopharynx regularly enough to explain our findings among controls. We could be detecting killed organisms within phagocytes and pneumococci processed for presentation to the immune system by antigen-presenting cells. It may be that blood stream invasion is relatively common in children but is normally eliminated by the immune system. In this model, invasive disease would only occur in the rare event that the immune system was unable to contain the infection. The high pneumococcal PCR positivity among children confirmed for bacterial pathogens other than pneumococcus suggests that illness itself might predispose to pneumococcal invasion from the nasopharynx or that pneumococcus acts as a copathogen.

The pneumococcal PCR assay in blood is not 100% specific in the diagnosis of pneumococcal pneumonia in all populations and should not be used as a diagnostic assay for clinical care without careful examination of test parameters, population by population. For the PERCH project, setting a quantitative threshold for positivity may be beneficial in predicting pneumococcal pneumonia. More work is needed to examine what happens when pneumococci breach the nasopharyngeal mucosa, how often this occurs, and potential host differences in handling such invasion events between different human populations. Although it is important to understand the pathophysiology, the fact remains that pneumococcal PCR on blood specimens is nonspecific in the diagnosis of pneumococcal pneumonia in children in low- and middle-income countries.

## Supplementary Data

Supplementary materials are available at *Clinical Infectious Diseases* online. Consisting of data provided by the authors to benefit the reader, the posted materials are not copyedited and are the sole responsibility of the authors, so questions or comments should be addressed to the corresponding author.

## Supplementary Material

Supplementary_Data_File

## Notes

***Author contributions.*** S. C. M. and M. D. K. led the analysis, interpreted results, and drafted the manuscript. J. A. G. S. and D. R. M. assisted with interpretation of results and drafting of the manuscript. N. L. W., D .E. P., and S. C. M. performed analyses and assisted with interpretation of results. M. D. K., J. A. G. S., H. C. B., W. A. B, D. R. F., L. L. H., S. R. C. H., K. L. K., O. S. L., S. A. M., K. L. O., D. M. T., R. A. K., and D. R. M. conceived and designed the study and supervised study conduct. S. C. M., D. E. P., P. V. A., D. A., M. A., C. B., A. N. D., A. J. D., L. P. G., M. M. H., A. K., G. K., S. M., R. M. D. P. M., J. M., S. N., C. P., S. O. S., B. T., and T. W. were involved in study conduct, data collection, and/or data management. S. Z. provided statistical guidance. All authors reviewed and approved the manuscript. S. C. M. and M. D. K. had full access to the data and had final responsibility for the decision to submit for publication.

***Acknowledgments.*** The authors would also like acknowledge members of the following groups who contributed to the study design, conduct, and/ analysis phases of PERCH (see Supplementary Materials for full list of names): Pneumonia Methods Working Group, PERCH Expert Group, PERCH Contributors, and the PERCH Chest Radiograph Reading Panel. This paper is published with the permission of the Director of the Kenya Medical Research Institute. We offer sincere thanks to the patients and families who participated in this study.

***Financial support.*** PERCH was supported by grant 48968 from the Bill & Melinda Gates Foundation to the International Vaccine Access Center, Department of International Health, Johns Hopkins Bloomberg School of Public Health. J. A. G. S. was supported by a clinical fellowship from The Wellcome Trust of Great Britain (098532).

***Supplement sponsorship.*** This article appears as part of the supplement “Pneumonia Etiology Research for Child Health (PERCH): Foundational Basis for the Primary Etiology Results,” sponsored by a grant from the Bill & Melinda Gates Foundation to the PERCH study of Johns Hopkins Bloomberg School of Public Health, Baltimore, Maryland.

***Potential conflicts of interest.*** M. D. K. has received funding for consultancies from Merck, Pfizer, and Novartis, and grant funding from Merck. L. L. H. has received grant funding from Pfizer and GlaxoSmithKline. K. L. K. has received grant funding from Merck Sharp & Dohme. S. A. M. has received honorarium for advisory board from the Bill & Melinda Gates Foundation, Pfizer, Medimmune and Novartis, institutional grants from GSK, Novartis, Pfizer, Minervax, and the Bill & Melinda Gates Foundation, and speakers bureau for Sanofi Pasteur and GSK. K. L. O. has received grant funding from GSK and Pfizer and participates on technical advisory boards for Merck, Sanofi Pasteur, PATH, Affinivax, and ClearPath. All other authors: No reported conflicts. All authors have submitted the ICMJE Form for Disclosure of Potential Conflicts of Interest. Conflicts that the editors consider relevant to the content of the manuscript have been disclosed.
