# Supplementary material for: Detection of Pneumococcal DNA in Blood by Polymerase Chain Reaction for Diagnosing Pneumococcal Pneumonia in Young Children From Low- and Middle-Income Countries
Source: Clin Infect Dis. 2017 May 29;64(Suppl 3):S347–56. doi: 10.1093/cid/cix145 (PMC5447841; doi:10.1093/cid/cix145)
Supplement: Supplementary_Data_File [file cix145_suppl_Supplementary_Data_File.docx]

**Supplementary Table 1.** Percent of PERCH enrolled children who were whole blood (WB) pneumococcal PCR positive by case and control groups, overall and by site

| **Site** | **MCPP cases** | | **MCPP BCx+ Cases** | | **Non-confirmed CXR-AC cases** | | **Non-confirmed CXR+ cases** | | **Non-confirmed cases** | | **Confirmed non-Spn bacterial case** | | **All cases** | | **All controls** | | **Non-RTI controls** | | **RTI controls** | |
| --- | --- | --- | --- | --- | --- | --- | --- | --- | --- | --- | --- | --- | --- | --- | --- | --- | --- | --- | --- | --- |
|  | **N** | **WB+**  **n (%)** | **N** | **WB+**  **n (%)** | **N** | **WB+**  **n (%)** | **N** | **WB+**  **n (%)** | **N** | **WB+**  **n (%)** | **N** | **WB+**  **n (%)** | **N** | **WB+**  **n (%)** | **N** | **WB+**  **n (%)** | **N** | **WB+**  **n (%)** | **N** | **WB+**  **n (%)** |
| All Sites | 56 | 36 (64.3) | 44 | 30 (68.2) | 853 | 83 (9.7) | 1745 | 127 (7.3) | 3832 | 243 (6.3) | 107 | 12 (11.2) | 3995 | 291 (7.3) | 4987 | 273 (5.5) | 3806 | 203 (5.3) | 1181 | 70 (5.9) |
| Kilifi, Kenya | 4 | 3 (75) | 4 | 3 (75.0) | 98 | 8 (8.2) | 239 | 15 ( 6.3) | 556 | 25 (4.5) | 6 | 3 (50.0) | 566 | 31 (5.5) | 751 | 48 (6.4) | 562 | 30 (5.3) | 189 | 18 (9.5) |
| Basse, The Gambia | 16 | 6 (37.5) | 10 | 5 (50.0) | 85 | 10 (11.8) | 253 | 20 (7.9) | 570 | 51 (8.9) | 16 | 4 (25.0) | 602 | 61 (10.1) | 608 | 47 (7.7) | 458 | 36 (7.9) | 150 | 11 (7.3) |
| Bamako, Mali | 24 | 19 (79.2) | 22 | 18 (81.8) | 128 | 16 (12.5) | 230 | 25 (10.9) | 619 | 56 (9.0) | 26 | 2 (7.7) | 669 | 77 (11.5) | 715 | 38 (5.3) | 419 | 22 (5.3) | 296 | 16 (5.4) |
| Lusaka, Zambia | 7 | 4 (57.1) | 7 | 4 (57.1) | 155 | 18 (11.6) | 221 | 22 (10.0) | 494 | 37 (7.5) | 23 | 2 (8.7) | 524 | 43 (8.2) | 603 | 31 (5.1) | 507 | 22 (4.3) | 96 | 9 (9.4) |
| Soweto, South Africa | 5 | 4 (80) | 1 | 0 (0.0) | 290 | 30 (10.3) | 500 | 44 (8.8) | 885 | 66 (7.5) | 27 | 1 (3.7) | 917 | 71 (7.7) | 963 | 98 (10.2) | 910 | 90 (9.9) | 53 | 8 (15.1) |
| Thailand | 0 | N/A | 0 | N/A | 41 | 0 (0.0) | 96 | 0 (0.0) | 218 | 3 (1.4) | 6 | 0 (0.0) | 224 | 3 (1.3) | 622 | 5 (0.8) | 378 | 1 (0.3) | 244 | 4 (1.6) |
| Bangladesh | 0 | N/A | 0 | N/A | 56 | 1 (1.8) | 206 | 1 (0.5) | 490 | 5 (1.0) | 3 | 0 (0.0) | 493 | 5 (1.0) | 725 | 6 (0.8) | 572 | 2 (0.3) | 153 | 4 (2.6) |

Abbreviations: MCPP, microbiologically confirmed pneumococcal pneumonia; BCx, blood culture; CXR, chest radiograph; CXR-AC, alveolar consolidation on CXR; RTI, controls with respiratory tract illness; Spn, pneumococcus; PCR, polymerase chain reaction; N/A, no children in this case group at the site.

MCPP defined as isolation of pneumococcus from blood culture, culture or PCR of lung aspirate or pleural fluid, or BinaxNOW antigen detection on pleural fluid.

CXR+ defined as radiographic evidence of pneumonia (consolidation and/or other infiltrates).

Confirmed non-Spn bacterial case was defined as a case with any non-Spn bacterial pathogen detected by blood culture, by lung aspirate culture or PCR, or by pleural fluid culture or PCR.

Results are presented graphically in Figure 1.

**Supplementary Tables 2A Whole blood pneumococcal PCR positivity by PERCH case-control group and clinical characteristics**

| **Kenya** | **All MCPP cases^a^ N=4** | | | **Non-Confirmed cases^b^ N=556** | | | **Non-Confirmed CXR+^c^ cases N=239** | | | **Non-Confirmed CXR-AC cases N=98** | | | **Confirmed non-pneu bacterial case^d^ N=6** | | | **All controls N=751** | | | **RTI controls N=189** | | | **Non-RTI controls N=562** | | |
| --- | --- | --- | --- | --- | --- | --- | --- | --- | --- | --- | --- | --- | --- | --- | --- | --- | --- | --- | --- | --- | --- | --- | --- | --- |
|  | **N** | **n (%) WB+** | **OR** | **N** | **n (%) WB+** | **OR** | **N** | **n (%) WB+** | **OR** | **N** | **n (%) WB+** | **OR** | **N** | **n (%) WB+** | **OR** | **N** | **n (%) WB+** | **OR** | **N** | **n (%) WB+** | **OR** | **N** | **n (%) WB+** | **OR** |
| **Overall** | **4** | **3 (75.0)** |  | **556** | **25 (4.5)** |  | **239** | **15 ( 6.3)** |  | **98** | **8 (8.2)** |  | **6** | **3 (50.0)** |  | **751** | **48 (6.4)** |  | **189** | **18 (9.5)** |  | **562** | **30 (5.3)** |  |
| **Age** |  | **--** |  |  | **P=.84** |  |  | **P=.64** |  |  | **P=.80** |  |  | **P=.55** |  |  | **P=.91** |  |  | **P=.84** |  |  | **P=.74** |  |
| **1-5 mos** | 0 | 0 (0.0) | ---- | 178 | 7 (3.9) | ---- | 72 | 3 (4.2) | ---- | 39 | 2 (5.1) | ---- | 3 | 2 (66.7) | ---- | 189 | 14 (7.4) | ---- | 46 | 4 (8.7) | ---- | 143 | 10 (7.0) | ---- |
| **6-11 mos** | 1 | 0 (0.0) | ---- | 114 | 5 (4.4) | 1.15 | 57 | 3 (5.3) | 1.28 | 14 | 1 (7.1) | 1.67 | 3 | 1 (33.3) | 0.36 | 167 | 11 (6.6) | 0.89 | 51 | 6 (11.8) | 1.35 | 116 | 5 (4.3) | 0.63 |
| **12-23 mos** | 1 | 1 (100) | ---- | 154 | 9 (5.8) | 1.49 | 73 | 7 (9.6) | 2.24 | 25 | 3 (12.0) | 2.33 | 0 | 0 (0.0) | ---- | 217 | 13 (6.0) | 0.8 | 57 | 4 (7.0) | 0.79 | 160 | 9 (5.6) | 0.8 |
| **24-59 mos** | 2 | 2 (100) | ---- | 110 | 4 (3.6) | 0.97 | 37 | 2 (5.4) | 1.4 | 20 | 2 (10.0) | 2.03 | 0 | 0 (0.0) | ---- | 178 | 10 (5.6) | 0.75 | 35 | 4 (11.4) | 1.35 | 143 | 6 (4.2) | 0.6 |
| **Gender** |  | **P=.50** |  |  | **P=.74** |  |  | **P=.52** |  |  | **P=.95** |  |  | **P=.56** |  |  | **P=.39** |  |  | **P=.55** |  |  | **P=.48** |  |
| **Female** | 2 | 2 (100) | 5 | 228 | 11 (4.8) | 1.15 | 108 | 8 (7.4) | 1.4 | 48 | 4 (8.3) | 1.04 | 5 | 2 (40.0) | 0.24 | 358 | 20 (5.6) | 0.77 | 97 | 8 (8.2) | 0.75 | 261 | 12 (4.6) | 0.77 |
| **Male** | 2 | 1 (50.0) | ---- | 328 | 14 (4.3) | ---- | 131 | 7 (5.3) | ---- | 50 | 4 (8.0) | ---- | 1 | 1 (100) | ---- | 392 | 28 (7.1) | ---- | 92 | 10 (10.9) | ---- | 300 | 18 (6.0) | ---- |
| **HIV infected** |  | **--** |  |  | **P=.61** |  |  | **P=.57** |  |  | **P=.71** |  |  | **--** |  |  | **P=.60** |  |  | **P=.66** |  |  | **P=.45** |  |
| **Yes** | 0 | 0 (0) | ---- | 4 | 0 (0.0) | 2.36 | 2 | 0 (0) | 2.95 | 2 | 0 (0.0) | 2.04 | 0 | 0 (0.0) | ---- | 2 | 0 (0.0) | 2.74 | 1 | 0 (0.0) | 2.76 | 1 | 0 (0.0) | 5.7 |
| **No** | 4 | .03 (75.0) | ---- | 500 | 22 (4.4) | ---- | 212 | 13 (6.1) | ---- | 83 | 7 (8.4) | ---- | 5 | 2 (40.0) | ---- | 638 | 43 (6.7) | ---- | 162 | 17 (10.5) | ---- | 476 | 26 (5.5) | ---- |
| **PCV vaccinated^e^** |  | **--** |  |  | **P=.31** |  |  | **P=.40** |  |  | **P=.41** |  |  | **P=.56** |  |  | **P=.29** |  |  | **P=.41** |  |  | **P=.28** |  |
| **Yes** | 4 | 3 (75.0) | ---- | 480 | 24 (5.0) | 2.38 | 215 | 15 (7.0) | 3.48 | 82 | 8 (9.8) | 3.54 | 5 | 2 (40.0) | 0.24 | 693 | 43 (6.2) | 0.57 | 179 | 16 (8.9) | 0.44 | 514 | 27 (5.3) | 0.52 |
| **No** | 0 | 0 (0.0) | ---- | 67 | 1 (1.5) | ---- | 22 | 0 (0.0) | ---- | 15 | 0 (0.0) | ---- | 1 | 1 (100) | ---- | 42 | 4 (9.5) | ---- | 7 | 1 (14.3) | ---- | 35 | 3 (8.6) | ---- |
| **Very severe pneumonia** |  | **P=.82** |  |  | **P=.09** |  |  | **P=.03** |  |  | **P=.11** |  |  | **P=.26** |  |  | **--** |  |  | **--** |  |  | **--** |  |
| **Yes** | 3 | 2 (66.7) | 0.56 | 282 | 17 (6.0) | 2.07 | 91 | 10 (11.0) | 3.36 | 44 | 6 (13.6) | 3.55 | 4 | 3 (75.0) | 11.7 | ---- | N/A | ---- | ---- | N/A | ---- | ---- | N/A | ---- |
| **No** | 1 | 1 (100) | ---- | 274 | 8 (2.9) | ---- | 148 | 5 (3.4) | ---- | 54 | 2 (3.7) | ---- | 2 | 0 (0.0) | ---- | ---- | N/A | ---- | ---- | N/A | ---- | ---- | N/A | ---- |
| **Prior antibiotic use^f^** |  | **P=.81** |  |  | **P=.26** |  |  | **P=.82** |  |  | **P=.90** |  |  | **P>.99** |  |  | **P=.02** |  |  | **P=.005** |  |  | **P=.56** |  |
| **Yes** | 1 | 1 (100) | 1.87 | 198 | 12 (6.1) | 1.6 | 83 | 6 (7.2) | 1.13 | 33 | 3 (9.1) | 1.1 | 2 | 1 (50.0) | 1 | 21 | 4 (19.0) | 3.9 | 5 | 3 (60.0) | 14.6 | 16 | 1 (6.3) | 1.69 |
| **No** | 3 | 2 (66.7) | ---- | 309 | 12 (3.9) | ---- | 136 | 9 (6.6) | ---- | 57 | 5 (8.8) | ---- | 4 | 2 (50.0) | ---- | 702 | 43 (6.1) | ---- | 176 | 15 (8.5) | ---- | 526 | 28 (5.3) | ---- |
| **NP culture positive for Pneumococcus** |  | **P=0.821** |  |  | **P=.09** |  |  | **P=.01** |  |  | **P=.049** |  |  | **P=.56** |  |  | **P=.03** |  |  | **P=.25** |  |  | **P=.16** |  |
| **Yes** | 3 | 2 (66.7) | 0.56 | 303 | 18 (5.9) | 2.12 | 131 | 14 (10.7) | 8.84 | 47 | 7 (14.9) | 6.23 | 5 | 2 (40.0) | 0.24 | 585 | 44 (7.5) | 2.93 | 166 | 18 (10.8) | 5.61 | 419 | 26 (6.2) | 2.07 |
| **No** | 1 | 1 (100) | ---- | 252 | 7 (2.8) | ---- | 108 | 1 (0.9) | ---- | 51 | 1 (2.0) | ---- | 1 | 1 (100) | ---- | 164 | 4 (2.4) | ---- | 22 | 0 (0.0) | ---- | 142 | 4 (2.8) | ---- |
| **Pneumococcus colonized (culture or PCR positive)** |  | **--** |  |  | **P=.07** |  |  | **P=.14** |  |  | **P=.25** |  |  | **--** |  |  | **P=.15** |  |  | **P=.57** |  |  | **P=.34** |  |
| **Yes** | 4 | 3 (75.0) | ---- | 434 | 24 (5.5) | 4.79 | 191 | 15 (7.9) | 8.52 | 76 | 8 (10.5) | 5.58 | 6 | 3 (50.0) | ---- | 662 | 46 (6.9) | 2.61 | 179 | 18 (10.1) | 2.4 | 483 | 28 (5.8) | 1.91 |
| **No** | 0 | 0 (0.0) | ---- | 121 | 1 (0.8) | ---- | 48 | 0 (0.0) | ---- | 22 | 0 (0.0) | ---- | 0 | 0 (0.0) | ---- | 88 | 2 (2.3) | ---- | 10 | 0 (0.0) | ---- | 78 | 2 (2.6) | ---- |
| **Pneumococcal NP/OP PCR density >6.9 log10 copies/mL** |  | **--** |  |  | **P=.27** |  |  | **P=.054** |  |  | **P=.02** |  |  | **P=.55** |  |  | **P=.60** |  |  | **P=.44** |  |  | **P=.93** |  |
| **Yes** | 0 | 0 (0.0) | ---- | 26 | 2 (7.7) | 2.2 | 10 | 2 (20.0) | 4.72 | 4 | 2 (50.0) | 13.6 | 3 | 1 (33.3) | 0.36 | 14 | 1 (7.1) | 1.61 | 7 | 1 (14.3) | 2.18 | 7 | 0 (0.0) | 1.14 |
| **No** | 4 | 3 (75.0) | ---- | 529 | 23 (4.3) | ---- | 229 | 13 (5.7) | ---- | 94 | 6 (6.4) | ---- | 3 | 2 (66.7) | ---- | 735 | 47 (6.4) | ---- | 182 | 17 (9.3) | ---- | 553 | 30 (5.4) | ---- |
| **NP/OP PCR positive for any virus** |  | **--** |  |  | **P=.91** |  |  | **P=.81** |  |  | **P=.89** |  |  | **P=.55** |  |  | **P=.76** |  |  | **P=.07** |  |  | **P=.13** |  |
| **Yes** | 4 | 3 (75.0) | ---- | 485 | 22 (4.5) | 0.94 | 213 | 14 (6.6) | 1.24 | 84 | 7 (8.3) | 0.87 | 3 | 1 (33.3) | 0.36 | 560 | 37 (6.6) | 1.11 | 147 | 11 (7.5) | 0.4 | 413 | 26 (6.3) | 2.18 |
| **No** | 0 | 0 (0.0) | ---- | 70 | 3 (4.3) | ---- | 26 | 1 (3.8) | ---- | 14 | 1 (7.1) | ---- | 3 | 2 (66.7) | ---- | 189 | 11 (5.8) | ---- | 42 | 7 (16.7) | ---- | 147 | 4 (2.7) | ---- |
| **Hypoxemia^g^** |  | **P=.29** |  |  | **P=.65** |  |  | **P=.79** |  |  | **P=.61** |  |  | **P=.55** |  |  | **--** |  |  | **--** |  |  | **--** |  |
| **Yes** | 1 | 0 (0) | 0.05 | 160 | 6 (3.8) | 0.81 | 89 | 5 (5.6) | 0.86 | 46 | 3 (6.5) | 0.69 | 3 | 1 (33.3) | 0.36 | ---- | N/A | ---- | ---- | N/A | ---- | ---- | N/A | ---- |
| **No** | 3 | 3 (100) | ---- | 393 | 19 (4.8) | ---- | 149 | 10 (6.7) | ---- | 52 | 5 (9.6) | ---- | 3 | 2 (66.7) | ---- | ---- | N/A | ---- | ---- | N/A | ---- | ---- | N/A | ---- |
| **Died in hospital** |  | **--** |  |  | **P=.56** |  |  | **P=.74** |  |  | **P=.79** |  |  | **P=.26** |  |  | **--** |  |  | **--** |  |  | **--** |  |
| **Yes** | 0 | 0 (0.0) | ---- | 23 | 0 (0.0) | 0.42 | 11 | 0 (0.0) | 0.6 | 7 | 0 (0.0) | 0.66 | 2 | 2 (100) | 11.7 | ---- | N/A | ---- | ---- | N/A | ---- | ---- | N/A | ---- |
| **No** | 4 | 3 (75.0) | ---- | 533 | 25 ( 4.7%) | ---- | 228 | 15 (6.6) | ---- | 91 | 8 (8.8) | ---- | 4 | 1 (25.0) | ---- | ---- | N/A | ---- | ---- | N/A | ---- | ---- | N/A | ---- |
| **CXR+** |  | **--** |  |  | **P=.10** |  |  | **--** |  |  | **--** |  |  | **--** |  |  | **--** |  |  | **--** |  |  | **--** |  |
| **Yes** | 4 | 3 (75.0) | ---- | 239 | 15 (6.3) | 2.06 | 239 | 15 (6.3) | ---- | 98 | 8 (8.2) | ---- | 5 | 2 (40.0) | ---- | ---- | N/A | ---- | ---- | N/A | ---- | ---- | N/A | ---- |
| **No** | 0 | 0 (0.0) | ---- | 261 | 8 (3.1) | ---- | 0 | 0 (0.0) | ---- | 0 | 0 (0.0) | ---- | 0 | 0 (0.0) | ---- | ---- | N/A | ---- | ---- | N/A | ---- | ---- | N/A | ---- |
| **Alveolar consolidation on CXR** |  | **P=.82** |  |  | **P=.06** |  |  | **P=.32** |  |  | **--** |  |  | **P=.45** |  |  | **--** |  |  | **--** |  |  | **--** |  |
| **Yes** | 3 | 2 (66.7) | 0.56 | 98 | 8 (8.2) | 2.35 | 98 | 8 (8.2) | 1.68 | 98 | 8 (8.2) | ---- | 4 | 1 (25.0) | 0.14 | ---- | N/A | ---- | ---- | N/A | ---- | ---- | N/A | ---- |
| **No** | 1 | 1 (100) | ---- | 402 | 15 (3.7) | ---- | 141 | 7 (5.0) | ---- | 0 | 0 (0.0) | ---- | 1 | 1 (100) | ---- | ---- | N/A | ---- | ---- | N/A | ---- | ---- | N/A | ---- |
| **WBC >15mm^3^** |  | **P=.82** |  |  | **P=.34** |  |  | **P=.49** |  |  | **P=.85** |  |  | **P=.12** |  |  | **--** |  |  | **--** |  |  | **--** |  |
| **Yes** | 3 | 2 (66.7) | 0.56 | 214 | 12 (5.6) | 1.47 | 106 | 8 (7.5) | 1.43 | 46 | 4 (8.7) | 1.14 | 3 | 0 (0.0) | 0.02 | ---- | N/A | ---- | ---- | N/A | ---- | ---- | N/A | ---- |
| **No** | 1 | 1 (100) | ---- | 334 | 13 (3.9) | ---- | 131 | 7 (5.3) | ---- | 52 | 4 (7.7) | ---- | 3 | 3 (100) | ---- | ---- | N/A | ---- | ---- | N/A | ---- | ---- | N/A | ---- |
| **CRP > 40mg/L** |  | **--** |  |  | **P=.75** |  |  | **P=.19** |  |  | **P=.14** |  |  | **--** |  |  | **--** |  |  | **--** |  |  | **--** |  |
| **Yes** | 4 | 3 (75.0) | ---- | 132 | 7 (5.3) | 1.16 | 61 | 6 (9.8) | 2.14 | 37 | 5 (13.5) | 4.23 | 4 | 1 (25.0) | ---- | ---- | N/A | ---- | ---- | N/A | ---- | ---- | N/A | ---- |
| **No** | 0 | 0 (0.0) | ---- | 295 | 14 (4.7) | ---- | 124 | 6 (4.8) | ---- | 38 | 1 (2.6) | ---- | 0 | 0 (0.0) | ---- | ---- | N/A | ---- | ---- | N/A | ---- | ---- | N/A | ---- |

N/A, data not collected for controls.

Abbreviations: MCPP, microbiologically confirmed pneumococcal pneumonia; WB, whole blood; PCV, pneumococcal conjugate vaccine; CXR, chest radiograph; CXR-AC, alveolar consolidation on CXR; WBC, white blood cells; CRP, C-reactive protein; HIV, human immunodeficiency virus; PCR, polymerase chain reaction; NP, nasopharyngeal; OP, oropharyngeal; RTI, respiratory tract illness.

^a^ MCPP defined as isolation of pneumococcus from blood culture, culture or PCR of lung aspirate or pleural fluid, or BinaxNOW antigen detection on pleural fluid.

^b^Non-confirmed cases defined as cases without isolation of bacteria from culture of blood, lung aspirate or pleural fluid, or PCR of lung aspirate or pleural fluid.

^c^ CXR+ defined as radiographic evidence of pneumonia (consolidation and/or other infiltrates).

^d^ Confirmed non-pneumococcal bacterial case was defined as a case with any non-pneumococcal bacterial pathogen detected by blood culture, by lung aspirate culture or PCR, or by pleural fluid culture or PCR.

^e^ PCV vaccinated defined as at least 1 dose.

^f^ Prior use of antibiotics defined as serum bioassay positive, antibiotic administration at the referral facility, or antibiotic administration prior to blood specimen collection at the study facility.

^g^ Hypoxemia was defined as <92% on room air (<90% at elevation, Zambia and South Africa) or a requirement for supplemental oxygen if a room air reading was not available.

**Supplementary Tables 2B Whole blood pneumococcal PCR positivity by PERCH case-control group and clinical characteristics**

| **The Gambia** | **All MCPP cases^a^ N=16** | | | **Non-Confirmed cases^b^ N=570** | | | **Non-Confirmed CXR+^c^ cases N=253** | | | **Non-Confirmed CXR-AC cases N=85** | | | **Confirmed non-pneu bacterial case^d^ N=16** | | | **All controls N=608** | | | **RTI controls N=150** | | | **Non-RTI controls N=458** | | |
| --- | --- | --- | --- | --- | --- | --- | --- | --- | --- | --- | --- | --- | --- | --- | --- | --- | --- | --- | --- | --- | --- | --- | --- | --- |
|  | **N** | **n (%) WB+** | **OR** | **N** | **n (%) WB+** | **OR** | **N** | **n (%) WB+** | **OR** | **N** | **n (%) WB+** | **OR** | **N** | **n (%) WB+** | **OR** | **N** | **n (%) WB+** | **OR** | **N** | **n (%) WB+** | **OR** | **N** | **n (%) WB+** | **OR** |
| **Overall** | **16** | **6 (37.5)** |  | **570** | **51 (8.9)** |  | **253** | **20 (7.9)** |  | **85** | **10 (11.8)** |  | **16** | **4 (25.0)** |  | **608** | **47 (7.7)** |  | **150** | **11 (7.3)** |  | **458** | **36 (7.9)** |  |
| **Age** |  | **P=.92** |  |  | **P=.35** |  |  | **P=.51** |  |  | **P=.29** |  |  | **P=.68** |  |  | **P=.44** |  |  | **P=.31** |  |  | **P=.89** |  |
| **1-5 mos** | 4 | 1 (25.0) | ---- | 237 | 21 (8.9) | ---- | 98 | 9 (9.2) | ---- | 33 | 3 (9.1) | ---- | 3 | 0 (0) | ---- | 184 | 18 (9.8) | ---- | 42 | 5 (11.9) | ---- | 142 | 13 (9.2) | ---- |
| **6-11 mos** | 4 | 2 (50.0) | 2.33 | 125 | 16 (12.8) | 1.52 | 62 | 7 (11.3) | 1.27 | 25 | 6 (24.0) | 2.9 | 3 | 0 (0) | 1 | 123 | 11 (8.9) | 0.92 | 33 | 4 (12.1) | 1.04 | 90 | 7 (7.8) | 0.86 |
| **12-23 mos** | 7 | 3 (42.9) | 1.82 | 115 | 8 (7.0) | 0.8 | 56 | 3 (5.4) | 0.62 | 18 | 1 (5.6) | 0.75 | 6 | 2 (33.3) | 3.89 | 169 | 9 (5.3) | 0.53 | 44 | 1 (2.3) | 0.24 | 125 | 8 (6.4) | 0.69 |
| **24-59 mos** | 1 | 0 (0) | 0.63 | 93 | 6 (6.5) | 0.75 | 37 | 1 (2.7) | 0.39 | 9 | 0 (0) | 0.46 | 4 | 2 (50.0) | 7 | 132 | 9 (6.8) | 0.69 | 31 | 1 (3.2) | 0.34 | 101 | 8 (7.9) | 0.87 |
| **Gender** |  | **P=.35** |  |  | **P=.28** |  |  | **P=.42** |  |  | **P=.60** |  |  | **P=.33** |  |  | **P=.84** |  |  | **P=.91** |  |  | **P=.76** |  |
| **Female** | 3 | 2 (66.7) | 3.52 | 221 | 16 (7.2) | 0.71 | 99 | 6 (6.1) | 0.67 | 33 | 3 (9.1) | 0.7 | 8 | 1 (12.5) | 0.31 | 289 | 23 (8.0) | 1.06 | 71 | 5 (7.0) | 0.94 | 218 | 18 (8.3) | 1.11 |
| **Male** | 13 | 4 (30.8) | ---- | 349 | 35 (10.0) | ---- | 154 | 14 (9.1) | ---- | 52 | 7 (13.5) | ---- | 8 | 3 (37.5) | ---- | 319 | 24 (7.5) | ---- | 79 | 6 (7.6) | ---- | 240 | 18 (7.5) | ---- |
| **HIV infected** |  | **--** |  |  | **P=.40** |  |  | **P=.17** |  |  | **P=.93** |  |  | **--** |  |  | **--** |  |  | **--** |  |  | **--** |  |
| **Yes** | 0 | 0 (0) | ---- | 7 | 1 (14.3) | 2.3 | 4 | 1 (25.0) | 4.57 | 3 | 0 (0) | 0.85 | 0 | 0 (0) | ---- | 0 | 0 (0) | ---- | 0 | 0 (0) | ---- | 0 | 0 (0) | ---- |
| **No** | 15 | 6 (40.0) | ---- | 486 | 44 (9.1) | ---- | 215 | 18 (8.4) | ---- | 72 | 10 (13.9) | ---- | 16 | 4 (25.0) | ---- | 608 | 47 (7.7) | ---- | 150 | 11 (7.3) | ---- | 458 | 36 (7.9) | ---- |
| **PCV vaccinated^e^** |  | **P=.55** |  |  | **P=.90** |  |  | **P=.75** |  |  | **P=.36** |  |  | **P=.70** |  |  | **P=.29** |  |  | **P=.33** |  |  | **P=.13** |  |
| **Yes** | 13 | 5 (38.5) | 3.24 | 409 | 39 (9.5) | 1.04 | 186 | 15 (8.1) | 0.85 | 61 | 9 (14.8) | 2.35 | 10 | 2 (20.0) | 2.06 | 498 | 41 (8.2) | 1.83 | 127 | 9 (7.1) | 0.47 | 371 | 32 (8.6) | 3.61 |
| **No** | 2 | 0 (0) | ---- | 134 | 12 (9.0) | ---- | 56 | 5 (8.9) | ---- | 20 | 1 (5.0) | ---- | 3 | 0 (0) | ---- | 73 | 3 (4.1) | ---- | 16 | 2 (12.5) | ---- | 57 | 1 (1.8) | ---- |
| **Very severe pneumonia** |  | **P=.14** |  |  | **P=.68** |  |  | **P=.36** |  |  | **P=.09** |  |  | **P=.33** |  |  | **--** |  |  | **--** |  |  | **--** |  |
| **Yes** | 11 | 6 (54.5) | 13 | 70 | 5 (7.1) | 0.82 | 26 | 3 (11.5) | 1.79 | 11 | 3 (27.3) | 3.71 | 8 | 1 (12.5) | 0.31 | ---- | N/A | ---- | ---- | N/A | ---- | ---- | N/A | ---- |
| **No** | 5 | 0 (0) | ---- | 500 | 46 (9.2) | ---- | 227 | 17 (7.5) | ---- | 74 | 7 (9.5) | ---- | 8 | 3 (37.5) | ---- | ---- | N/A | ---- | ---- | N/A | ---- | ---- | N/A | ---- |
| **Prior antibiotic use^f^** |  | **P=.27** |  |  | **P=.63** |  |  | **P=.65** |  |  | **P=.83** |  |  | **P=.72** |  |  | **P=.57** |  |  | **P=.57** |  |  | **--** |  |
| **Yes** | 3 | 2 (66.7) | 4.52 | 49 | 5 (10.2) | 1.26 | 25 | 1 (4.0) | 0.67 | 13 | 1 (7.7) | 0.81 | 3 | 1 (33.3) | 1.63 | 1 | 0 (0) | 3.71 | 1 | 0 (0) | 3.77 | 0 | 0 (0) | ---- |
| **No** | 12 | 3 (25.0) | ---- | 476 | 42 (8.8) | ---- | 207 | 17 (8.2) | ---- | 65 | 8 (12.3) | ---- | 12 | 3 (25.0) | ---- | 581 | 47 (8.1) | ---- | 142 | 11 (7.7) | ---- | 439 | 36 (8.2) | ---- |
| **NP culture positive for Pneumococcus** |  | **P=.82** |  |  | **P=.39** |  |  | **P=.69** |  |  | **P=.88** |  |  | **P=.87** |  |  | **P=.21** |  |  | **P=.051** |  |  | **P=.53** |  |
| **Yes** | 14 | 5 (35.7) | 1.74 | 478 | 41 (8.6) | 0.73 | 206 | 16 (7.8) | 0.8 | 71 | 9 (12.7) | 1.16 | 9 | 2 (22.2) | 1.22 | 529 | 39 (7.4) | 0.6 | 140 | 9 (6.4) | 0.19 | 389 | 30 (7.7) | 0.75 |
| **No** | 1 | 0 (0) | ---- | 90 | 10 (11.1) | ---- | 45 | 4 (8.9) | ---- | 12 | 1 (8.3) | ---- | 6 | 1 (16.7) | ---- | 71 | 8 (11.3) | ---- | 8 | 2 (25.0) | ---- | 63 | 6 (9.5) | ---- |
| **Pneumococcus colonized (culture or PCR positive)** |  | **--** |  |  | **P=.80** |  |  | **P=.33** |  |  | **P=.66** |  |  | **P=.76** |  |  | **P=.04** |  |  | **P=.10** |  |  | **P=.11** |  |
| **Yes** | 15 | 5 (33.3) | ---- | 524 | 47 (9.0) | 0.87 | 229 | 20 (8.7) | 4.21 | 77 | 10 (13.0) | 2.02 | 12 | 3 (25.0) | 1.84 | 571 | 42 (7.4) | 0.36 | 146 | 10 (6.8) | 0.13 | 425 | 32 (7.5) | 0.41 |
| **No** | 0 | 0 (0) | ---- | 43 | 4 (9.3) | ---- | 21 | 0 (0) | ---- | 6 | 0 (0) | ---- | 2 | 0 (0) | ---- | 29 | 5 (17.2) | ---- | 3 | 1 (33.3) | ---- | 26 | 4 (15.4) | ---- |
| **Pneumococcal NP/OP PCR density >6.9 log10 copies/mL** |  | **P=.55** |  |  | **P=.81** |  |  | **P=.29** |  |  | **P=.66** |  |  | **P=.43** |  |  | **P=.21** |  |  | **P=.19** |  |  | **P=.41** |  |
| **Yes** | 10 | 4 (40.0) | 2.08 | 84 | 8 (9.5) | 1.1 | 42 | 5 (11.9) | 1.76 | 21 | 3 (14.3) | 1.37 | 4 | 0 (0) | 0.24 | 49 | 6 (12.2) | 1.77 | 13 | 2 (15.4) | 2.87 | 36 | 4 (11.1) | 1.56 |
| **No** | 5 | 1 (20.0) | ---- | 474 | 43 (9.1) | ---- | 201 | 15 (7.5) | ---- | 61 | 7 (11.5) | ---- | 10 | 3 (30.0) | ---- | 532 | 41 (7.7) | ---- | 134 | 9 (6.7) | ---- | 398 | 32 (8.0) | ---- |
| **NP/OP PCR positive for any virus** |  | **--** |  |  | **P=.87** |  |  | **P=.97** |  |  | **P=.53** |  |  | **P=.76** |  |  | **P=.44** |  |  | **P=.25** |  |  | **P=.73** |  |
| **Yes** | 15 | 5 (33.3) | ---- | 518 | 48 (9.3) | 1.1 | 226 | 19 (8.4) | 1.03 | 76 | 9 (11.8) | 0.52 | 12 | 3 (25.0) | 1.84 | 456 | 35 (7.7) | 0.76 | 123 | 8 (6.5) | 0.45 | 333 | 27 (8.1) | 0.87 |
| **No** | 0 | 0 (0) | ---- | 40 | 3 (7.5) | ---- | 17 | 1 (5.9) | ---- | 6 | 1 (16.7) | ---- | 2 | 0 (0) | ---- | 125 | 12 (9.6) | ---- | 24 | 3 (12.5) | ---- | 101 | 9 (8.9) | ---- |
| **Hypoxemia^g^** |  | **P=.22** |  |  | **P=.14** |  |  | **P=.26** |  |  | **P=.39** |  |  | **P=.38** |  |  | **--** |  |  | **--** |  |  | **--** |  |
| **Yes** | 2 | 2 (100) | 11.7 | 39 | 0 (0) | 0.12 | 26 | 0 (0) | 0.19 | 11 | 0 (0) | 0.27 | 4 | 0 (0) | 0.21 | ---- | N/A | ---- | ---- | N/A | ---- | ---- | N/A | ---- |
| **No** | 14 | 4 (28.6) | ---- | 530 | 51 (9.6) | ---- | 227 | 20 (8.8) | ---- | 74 | 10 (13.5) | ---- | 12 | 4 (33.3) | ---- | ---- | N/A | ---- | ---- | N/A | ---- | ---- | N/A | ---- |
| **Died in hospital** |  | **P=.15** |  |  | **P=.84** |  |  | **P=.32** |  |  | **P=.40** |  |  | **P=.91** |  |  | **--** |  |  | **--** |  |  | **--** |  |
| **Yes** | 4 | 3 (75.0) | 6.33 | 13 | 1 (7.7) | 1.21 | 7 | 1 (14.3) | 2.69 | 5 | 1 (20.0) | 2.51 | 4 | 1 (25.0) | 1.16 | ---- | N/A | ---- | ---- | N/A | ---- | ---- | N/A | ---- |
| **No** | 12 | 3 (25.0) | ---- | 557 | 50 (9.0) | ---- | 246 | 19 (7.7) | ---- | 80 | 9 (11.3) | ---- | 12 | 3 (25.0) | ---- | ---- | N/A | ---- | ---- | N/A | ---- | ---- | N/A | ---- |
| **CXR+** |  | **P=.24** |  |  | **P=.51** |  |  | **--** |  |  | **--** |  |  | **P=.88** |  |  | **--** |  |  | **--** |  |  | **--** |  |
| **Yes** | 13 | 4 (30.8) | 0.09 | 253 | 20 (7.9) | 0.82 | 253 | 20 (7.9) | ---- | 85 | 10 (11.8) | ---- | 8 | 2 (25.0) | 0.85 | ---- | N/A | ---- | ---- | N/A | ---- | ---- | N/A | ---- |
| **No** | 2 | 2 (100) | ---- | 293 | 28 (9.6) | ---- | 0 | 0 (0) | ---- | 0 | 0 (0) | ---- | 7 | 2 (28.6) | ---- | ---- | N/A | ---- | ---- | N/A | ---- | ---- | N/A | ---- |
| **Alveolar consolidation on CXR** |  | **P=.17** |  |  | **P=.25** |  |  | **P=.11** |  |  | **--** |  |  | **P=.79** |  |  | **--** |  |  | **--** |  |  | **--** |  |
| **Yes** | 11 | 3 (27.3) | 0.18 | 85 | 10 (11.8) | 1.53 | 85 | 10 (11.8) | 2.1 | 85 | 10 (11.8) | ---- | 5 | 1 (20.0) | 0.71 | ---- | N/A | ---- | ---- | N/A | ---- | ---- | N/A | ---- |
| **No** | 4 | 3 (75.0) | ---- | 461 | 38 (8.2) | ---- | 168 | 10 (6.0) | ---- | 0 | 0 (0) | ---- | 10 | 3 (30.0) | ---- | ---- | N/A | ---- | ---- | N/A | ---- | ---- | N/A | ---- |
| **WBC >15mm^3^** |  | **P=.32** |  |  | **P=.69** |  |  | **P=.54** |  |  | **P=.16** |  |  | **P=.52** |  |  | **--** |  |  | **--** |  |  | **--** |  |
| **Yes** | 7 | 3 (42.9) | 3.37 | 153 | 12 (7.8) | 0.86 | 76 | 6 (7.9) | 1.43 | 26 | 4 (15.4) | 3.24 | 9 | 2 (22.2) | 0.47 | ---- | N/A | ---- | ---- | N/A | ---- | ---- | N/A | ---- |
| **No** | 7 | 1 (14.3) | ---- | 274 | 25 (9.1) | ---- | 106 | 6 (5.7) | ---- | 42 | 2 (4.8) | ---- | 5 | 2 (40.0) | ---- | ---- | N/A | ---- | ---- | N/A | ---- | ---- | N/A | ---- |
| **CRP >40mg/L** |  | **P=.53** |  |  | **P=.40** |  |  | **P=.24** |  |  | **P=.57** |  |  | **P=.68** |  |  | **--** |  |  | **--** |  |  | **--** |  |
| **Yes** | 10 | 3 (30.0) | 3.27 | 113 | 11 (9.7) | 1.4 | 73 | 7 (9.6) | 2.07 | 33 | 3 (9.1) | 1.8 | 9 | 2 (22.2) | 0.56 | ---- | N/A | ---- | ---- | N/A | ---- | ---- | N/A | ---- |
| **No** | 3 | 0 (0) | ---- | 248 | 18 (7.3) | ---- | 86 | 4 (4.7) | ---- | 24 | 1 (4.2) | ---- | 3 | 1 (33.3) | ---- | ---- | N/A | ---- | ---- | N/A | ---- | ---- | N/A | ---- |

N/A, data not collected for controls.

Abbreviations: MCPP, microbiologically confirmed pneumococcal pneumonia; WB, whole blood; PCV, pneumococcal conjugate vaccine; CXR, chest radiograph; CXR-AC, alveolar consolidation on CXR; WBC, white blood cells; CRP, C-reactive protein; HIV, human immunodeficiency virus; PCR, polymerase chain reaction; NP, nasopharyngeal; OP, oropharyngeal; RTI, respiratory tract illness.

^a^ MCPP defined as isolation of pneumococcus from blood culture, culture or PCR of lung aspirate or pleural fluid, or BinaxNOW antigen detection on pleural fluid.

^b^Non-confirmed cases defined as cases without isolation of bacteria from culture of blood, lung aspirate or pleural fluid, or PCR of lung aspirate or pleural fluid.

^c^ CXR+ defined as radiographic evidence of pneumonia (consolidation and/or other infiltrates).

^d^ Confirmed non-pneumococcal bacterial case was defined as a case with any non-pneumococcal bacterial pathogen detected by blood culture, by lung aspirate culture or PCR, or by pleural fluid culture or PCR.

^e^ PCV vaccinated defined as at least 1 dose.

^f^ Prior use of antibiotics defined as serum bioassay positive, antibiotic administration at the referral facility, or antibiotic administration prior to blood specimen collection at the study facility.

^g^ Hypoxemia was defined as <92% on room air (<90% at elevation, Zambia and South Africa) or a requirement for supplemental oxygen if a room air reading was not available.

**Supplementary Tables 2C Whole blood pneumococcal PCR positivity by PERCH case-control group and clinical characteristics**

| **Mali** | **All MCPP cases^a^ N=24** | | | **Non-Confirmed cases^b^ N=619** | | | **Non-Confirmed CXR+^c^ cases N=230** | | | **Non-Confirmed CXR-AC cases N=128** | | | **Confirmed non-pneu bacterial case^d^ N=26** | | | **All controls N=715** | | | **RTI controls N=296** | | | **Non-RTI controls N=419** | | |
| --- | --- | --- | --- | --- | --- | --- | --- | --- | --- | --- | --- | --- | --- | --- | --- | --- | --- | --- | --- | --- | --- | --- | --- | --- |
|  | **N** | **n (%) WB+** | **OR** | **N** | **n (%) WB+** | **OR** | **N** | **n (%) WB+** | **OR** | **N** | **n (%) WB+** | **OR** | **N** | **n (%) WB+** | **OR** | **N** | **n (%) WB+** | **OR** | **N** | **n (%) WB+** | **OR** | **N** | **n (%) WB+** | **OR** |
| **Overall** | **24** | **19 (79.2)** |  | **619** | **56 (9.0)** |  | **230** | **25 (10.9)** |  | **128** | **16 (12.5)** |  | **26** | **2 (7.7)** |  | **715** | **38 (5.3)** |  | **296** | **16 (5.4)** |  | **419** | **22 (5.3)** |  |
| **Age** |  | **P=.60** |  |  | **P=.18** |  |  | **P=.32** |  |  | **P=.10** |  |  | **P=.75** |  |  | **P=.40** |  |  | **P>.99** |  |  | **P=.17** |  |
| **1-5 mos** | 6 | 5 (83.3) | ---- | 289 | 20 ( 6.9) | ---- | 97 | 10 (10.3) | ---- | 52 | 5 (9.6) | ---- | 10 | 0 (0) | ---- | 243 | 14 (5.8) | ---- | 72 | 4 (5.6) | ---- | 171 | 10 (5.8) | ---- |
| **6-11 mos** | 5 | 5 (100) | 3 | 139 | 18 (12.9) | 2 | 57 | 5 (8.8) | 0.87 | 37 | 3 (8.1) | 0.88 | 6 | 1 (16.7) | 5.73 | 184 | 8 (4.3) | 0.76 | 82 | 4 (4.9) | 0.87 | 102 | 4 (3.9) | 0.7 |
| **12-23 mos** | 4 | 2 (50.0) | 0.27 | 130 | 14 (10.8) | 1.64 | 58 | 10 (17.2) | 1.8 | 29 | 8 (27.6) | 3.41 | 6 | 1 (16.7) | 5.73 | 163 | 6 (3.7) | 0.65 | 86 | 5 (5.8) | 1.03 | 77 | 1 (1.3) | 0.3 |
| **24-59 mos** | 9 | 7 (77.8) | 0.82 | 61 | 4 (6.6) | 1.03 | 18 | 0 (0) | 0.23 | 10 | 0 (0) | 0.41 | 4 | 0 (0) | 2.33 | 125 | 10 (8.0) | 1.44 | 56 | 3 (5.4) | 1 | 69 | 7 (10.1) | 1.85 |
| **Gender** |  | **P=.97** |  |  | **P=.21** |  |  | **P=.70** |  |  | **P=.59** |  |  | **P=.33** |  |  | **P=.37** |  |  | **P=.40** |  |  | **P=.66** |  |
| **Female** | 14 | 11 (78.6) | 0.97 | 271 | 20 (7.4) | 0.7 | 92 | 9 (9.8) | 0.84 | 56 | 8 (14.3) | 1.33 | 14 | 2 (14.3) | 5 | 362 | 22 (6.1) | 1.35 | 153 | 10 (6.5) | 1.55 | 209 | 12 (5.7) | 1.21 |
| **Male** | 10 | 8 (80.0) | ---- | 348 | 36 (10.3) | ---- | 138 | 16 (11.6) | ---- | 72 | 8 (11.1) | ---- | 12 | 0 (0) | ---- | 353 | 16 (4.5) | ---- | 143 | 6 (4.2) | ---- | 210 | 10 (4.8) | ---- |
| **HIV infected** |  | **P=.52** |  |  | **P=.38** |  |  | **P=.17** |  |  | **P=.18** |  |  | **P=.17** |  |  | **--** |  |  | **--** |  |  | **--** |  |
| **Yes** | 6 | 6 (100) | 3.1 | 13 | 2 (15.4) | 1.93 | 8 | 2 (25.0) | 3.16 | 7 | 2 (28.6) | 3.23 | 2 | 1 (50.0) | 9.67 | 0 | 0 (0) | ---- | 0 | 0 (0) | ---- | 0 | 0 (0) | ---- |
| **No** | 12 | 10 (83.3) | ---- | 419 | 42 (10.0) | ---- | 151 | 16 (10.6) | ---- | 84 | 10 (11.9) | ---- | 15 | 1 (6.7) | ---- | 281 | 13 (4.6) | ---- | 133 | 6 (4.5) | ---- | 148 | 7 (4.7) | ---- |
| **PCV vaccinated^e^** |  | **P=.16** |  |  | **P=.98** |  |  | **P=.85** |  |  | **P=.94** |  |  | **P=.55** |  |  | **P=.42** |  |  | **P=.49** |  |  | **P=.70** |  |
| **Yes** | 15 | 10 (66.7) | 0.1 | 441 | 40 (9.1) | 0.99 | 164 | 18 (11.0) | 0.92 | 90 | 12 (13.3) | 1.04 | 17 | 2 (11.8) | 2.74 | 479 | 28 (5.8) | 1.35 | 194 | 12 (6.2) | 1.48 | 285 | 16 (5.6) | 1.2 |
| **No** | 9 | 9 (100) | ---- | 167 | 15 (9.0) | ---- | 61 | 7 (11.5) | ---- | 33 | 4 (12.1) | ---- | 8 | 0 (0) | ---- | 234 | 10 (4.3) | ---- | 101 | 4 (4.0) | ---- | 133 | 6 (4.5) | ---- |
| **Very severe pneumonia** |  | **P=.39** |  |  | **P=.12** |  |  | **P=.22** |  |  | **P=.38** |  |  | **P=.51** |  |  | **--** |  |  | **--** |  |  | **--** |  |
| **Yes** | 14 | 12 (85.7) | 2.33 | 316 | 23 (7.3) | 0.65 | 92 | 7 (7.6) | 0.57 | 46 | 4 (8.7) | 0.6 | 17 | 2 (11.8) | 3.06 | ---- | N/A | ---- | ---- | N/A | ---- | ---- | N/A | ---- |
| **No** | 10 | 7 (70.0) | ---- | 303 | 33 (10.9) | ---- | 138 | 18 (13.0) | ---- | 82 | 12 (14.6) | ---- | 9 | 0 (0) | ---- | ---- | N/A | ---- | ---- | N/A | ---- | ---- | N/A | ---- |
| **Prior antibiotic use^f^** |  | **P=.52** |  |  | **P=.78** |  |  | **P=.04** |  |  | **P=.10** |  |  | **P=.17** |  |  | **P=.03** |  |  | **P=.90** |  |  | **P=.007** |  |
| **Yes** | 3 | 2 (66.7) | 0.43 | 160 | 15 (9.4) | 1.09 | 53 | 10 (18.9) | 2.51 | 28 | 6 (21.4) | 2.49 | 10 | 2 (20.0) | 9.7 | 19 | 3 (15.8) | 3.81 | 6 | 0 (0) | 1.23 | 13 | 3 (23.1) | 6.39 |
| **No** | 21 | 17 (81.0) | ---- | 455 | 40 (8.8) | ---- | 176 | 15 (8.5) | ---- | 100 | 10 (10.0) | ---- | 16 | 0 (0) | ---- | 672 | 35 (5.2) | ---- | 280 | 16 (5.7) | ---- | 392 | 19 (4.8) | ---- |
| **NP culture positive for Pneumococcus** |  | **P=.52** |  |  | **P=.86** |  |  | **P=.65** |  |  | **P=.66** |  |  | **P=.42** |  |  | **P=.98** |  |  | **P=.31** |  |  | **P=.51** |  |
| **Yes** | 21 | 17 (81.0) | 2.33 | 264 | 25 (9.5) | 1.05 | 104 | 13 (12.5) | 1.21 | 63 | 9 (14.3) | 1.27 | 10 | 0 (0) | 0.26 | 409 | 20 (4.9) | 0.99 | 199 | 9 (4.5) | 0.59 | 210 | 11 (5.2) | 1.37 |
| **No** | 3 | 2 (66.7) | ---- | 320 | 29 (9.1) | ---- | 114 | 12 (10.5) | ---- | 61 | 7 (11.5) | ---- | 15 | 2 (13.3) | ---- | 267 | 13 (4.9) | ---- | 82 | 6 (7.3) | ---- | 185 | 7 (3.8) | ---- |
| **Pneumococcus colonized (culture or PCR positive)** |  | **--** |  |  | **P=.36** |  |  | **P=.28** |  |  | **P=.10** |  |  | **P=.63** |  |  | **P=.87** |  |  | **P=.85** |  |  | **P=.88** |  |
| **Yes** | 24 | 19 (79.2) | ---- | 464 | 46 (9.9) | 1.39 | 164 | 21 (12.8) | 1.81 | 96 | 16 (16.7) | 11.7 | 18 | 2 (11.1) | 2.27 | 579 | 31 (5.4) | 1.07 | 257 | 14 (5.4) | 0.87 | 322 | 17 (5.3) | 1.09 |
| **No** | 0 | 0 (0) | ---- | 141 | 10 (7.1) | ---- | 58 | 4 (6.9) | ---- | 28 | 0 (0) | ---- | 7 | 0 (0) | ---- | 127 | 6 (4.7) | ---- | 38 | 2 (5.3) | ---- | 89 | 4 (4.5) | ---- |
| **Pneumococcal NP/OP PCR density >6.9 log10 copies/mL** |  | **P=.52** |  |  | **P=.41** |  |  | **P=.12** |  |  | **P=.07** |  |  | **P=.90** |  |  | **P=.46** |  |  | **P=.25** |  |  | **P=.73** |  |
| **Yes** | 21 | 17 (81.0) | 2.33 | 140 | 15 (10.7) | 1.3 | 54 | 9 (16.7) | 2.01 | 32 | 7 (21.9) | 2.65 | 12 | 1 (8.3) | 1.17 | 112 | 4 (3.6) | 0.68 | 59 | 1 (1.7) | 0.37 | 53 | 3 (5.7) | 1.23 |
| **No** | 3 | 2 (66.7) | ---- | 476 | 41 (8.6) | ---- | 174 | 16 ( 9.2) | ---- | 94 | 9 (9.6) | ---- | 14 | 1 (7.1) | ---- | 602 | 34 (5.6) | ---- | 236 | 15 (6.4) | ---- | 366 | 19 (5.2) | ---- |
| **NP/OP PCR positive for any virus** |  | **P=.96** |  |  | **P=.17** |  |  | **P=.03** |  |  | **P=.01** |  |  | **P=.26** |  |  | **P=.97** |  |  | **P=.68** |  |  | **P=.69** |  |
| **Yes** | 23 | 18 (78.3) | 1.14 | 566 | 49 (8.7) | 0.55 | 209 | 20 (9.6) | 0.29 | 116 | 12 (10.3) | 0.17 | 21 | 1 (4.8) | 0.22 | 557 | 30 (5.4) | 1.02 | 241 | 14 (5.8) | 1.34 | 316 | 16 (5.1) | 0.82 |
| **No** | 1 | 1 (100) | ---- | 50 | 7 (14.0) | ---- | 19 | 5 (26.3) | ---- | 10 | 4 (40.0) | ---- | 5 | 1 (20.0) | ---- | 157 | 8 (5.1) | ---- | 54 | 2 (3.7) | ---- | 103 | 6 (5.8) | ---- |
| **Hypoxemia^g^** |  | **P=.39** |  |  | **P=.74** |  |  | **P=.09** |  |  | **P=.11** |  |  | **P=.29** |  |  | **--** |  |  | **--** |  |  | **--** |  |
| **Yes** | 14 | 12 (85.7) | 2.33 | 290 | 25 (8.6) | 0.91 | 130 | 10 (7.7) | 0.48 | 73 | 6 (8.2) | 0.42 | 13 | 2 (15.4) | 5.87 | ---- | N/A | ---- | ---- | N/A | ---- | ---- | N/A | ---- |
| **No** | 10 | 7 (70.0) | ---- | 329 | 31 (9.4) | ---- | 100 | 15 (15.0) | ---- | 55 | 10 (18.2) | ---- | 13 | 0 (0) | ---- | ---- | N/A | ---- | ---- | N/A | ---- | ---- | N/A | ---- |
| **Died in hospital** |  | **P=.69** |  |  | **P=.47** |  |  | **P=.76** |  |  | **P=.54** |  |  | **P=.61** |  |  | **--** |  |  | **--** |  |  | **--** |  |
| **Yes** | 8 | 6 (75.0) | 0.67 | 83 | 9 (10.8) | 1.31 | 26 | 3 (11.5) | 1.21 | 19 | 3 (15.8) | 1.52 | 9 | 1 (11.1) | 1.94 | ---- | N/A | ---- | ---- | N/A | ---- | ---- | N/A | ---- |
| **No** | 16 | 13 (81.3) | ---- | 536 | 47 (8.8) | ---- | 204 | 22 (10.8) | ---- | 109 | 13 (11.9) | ---- | 17 | 1 (5.9) | ---- | ---- | N/A | ---- | ---- | N/A | ---- | ---- | N/A | ---- |
| **CXR+** |  | **P=.57** |  |  | **P=.23** |  |  | **--** |  |  | **--** |  |  | **P=.62** |  |  | **--** |  |  | **--** |  |  | **--** |  |
| **Yes** | 11 | 8 (72.7) | 0.35 | 230 | 25 (10.9) | 1.45 | 230 | 25 (10.9) | ---- | 128 | 16 (12.5) | ---- | 11 | 1 (9.1) | 2.43 | ---- | N/A | ---- | ---- | N/A | ---- | ---- | N/A | ---- |
| **No** | 3 | 3 (100) | ---- | 259 | 20 (7.7) | ---- | 0 | 0 (0) | ---- | 0 | 0 (0) | ---- | 8 | 0 (0) | ---- | ---- | N/A | ---- | ---- | N/A | ---- | ---- | N/A | ---- |
| **Alveolar consolidation on CXR** |  | **P>.99** |  |  | **P=.12** |  |  | **P=.40** |  |  | **--** |  |  | **P=.62** |  |  | **--** |  |  | **--** |  |  | **--** |  |
| **Yes** | 9 | 7 (77.8) | 1 | 128 | 16 (12.5) | 1.65 | 128 | 16 (12.5) | 1.44 | 128 | 16 (12.5) | ---- | 8 | 0 (0) | 0.41 | ---- | N/A | ---- | ---- | N/A | ---- | ---- | N/A | ---- |
| **No** | 5 | 4 (80.0) | ---- | 361 | 29 (8.0) | ---- | 102 | 9 (8.8) | ---- | 0 | 0 (0) | ---- | 11 | 1 (9.1) | ---- | ---- | N/A | ---- | ---- | N/A | ---- | ---- | N/A | ---- |
| **WBC >15mm^3^** |  | **P=.28** |  |  | **P=.99** |  |  | **P=.38** |  |  | **P=.96** |  |  | **P=.66** |  |  | **--** |  |  | **--** |  |  | **--** |  |
| **Yes** | 9 | 6 (66.7) | 0.34 | 146 | 13 (8.9) | 1 | 65 | 5 (7.7) | 0.64 | 40 | 5 (12.5) | 1.03 | 7 | 0 (0) | 0.47 | ---- | N/A | ---- | ---- | N/A | ---- | ---- | N/A | ---- |
| **No** | 15 | 13 (86.7) | ---- | 471 | 43 (9.1) | ---- | 164 | 20 (12.2) | ---- | 87 | 11 (12.6) | ---- | 19 | 2 (10.5) | ---- | ---- | N/A | ---- | ---- | N/A | ---- | ---- | N/A | ---- |
| **CRP >40mg/L** |  | **P=.70** |  |  | **P=.06** |  |  | **P=.93** |  |  | **P=.34** |  |  | **P=.25** |  |  | **--** |  |  | **--** |  |  | **--** |  |
| **Yes** | 16 | 13 (81.3) | 1.65 | 162 | 20 (12.3) | 1.76 | 81 | 9 (11.1) | 1.04 | 50 | 8 (16.0) | 1.71 | 18 | 1 (5.6) | 0.2 | ---- | N/A | ---- | ---- | N/A | ---- | ---- | N/A | ---- |
| **No** | 4 | 3 (75.0) | ---- | 377 | 28 (7.4) | ---- | 120 | 13 (10.8) | ---- | 61 | 6 ( 9.8) | ---- | 4 | 1 (25.0) | ---- | ---- | N/A | ---- | ---- | N/A | ---- | ---- | N/A | ---- |

N/A, data not collected for controls.

Abbreviations: MCPP, microbiologically confirmed pneumococcal pneumonia; WB, whole blood; PCV, pneumococcal conjugate vaccine; CXR, chest radiograph; CXR-AC, alveolar consolidation on CXR; WBC, white blood cells; CRP, C-reactive protein; HIV, human immunodeficiency virus; PCR, polymerase chain reaction; NP, nasopharyngeal; OP, oropharyngeal; RTI, respiratory tract illness.

^a^ MCPP defined as isolation of pneumococcus from blood culture, culture or PCR of lung aspirate or pleural fluid, or BinaxNOW antigen detection on pleural fluid.

^b^Non-confirmed cases defined as cases without isolation of bacteria from culture of blood, lung aspirate or pleural fluid, or PCR of lung aspirate or pleural fluid.

^c^ CXR+ defined as radiographic evidence of pneumonia (consolidation and/or other infiltrates).

^d^ Confirmed non-pneumococcal bacterial case was defined as a case with any non-pneumococcal bacterial pathogen detected by blood culture, by lung aspirate culture or PCR, or by pleural fluid culture or PCR.

^e^ PCV vaccinated defined as at least 1 dose.

^f^ Prior use of antibiotics defined as serum bioassay positive, antibiotic administration at the referral facility, or antibiotic administration prior to blood specimen collection at the study facility.

^g^ Hypoxemia was defined as <92% on room air (<90% at elevation, Zambia and South Africa) or a requirement for supplemental oxygen if a room air reading was not available.

**Supplementary Tables 2D Whole blood pneumococcal PCR positivity by PERCH case-control group and clinical characteristics**

| **Zambia** | **All MCPP cases^a^ N=7** | | | **Non-Confirmed cases^b^ N=494** | | | **Non-Confirmed CXR+^c^ cases N=221** | | | **Non-Confirmed CXR-AC cases N=155** | | | **Confirmed non-pneu bacterial case^d^ N=23** | | | **All controls N=603** | | | **RTI controls N=96** | | | **Non-RTI controls N=507** | | |
| --- | --- | --- | --- | --- | --- | --- | --- | --- | --- | --- | --- | --- | --- | --- | --- | --- | --- | --- | --- | --- | --- | --- | --- | --- |
|  | **N** | **n (%) WB+** | **OR** | **N** | **n (%) WB+** | **OR** | **N** | **n (%) WB+** | **OR** | **N** | **n (%) WB+** | **OR** | **N** | **n (%) WB+** | **OR** | **N** | **n (%) WB+** | **OR** | **N** | **n (%) WB+** | **OR** | **N** | **n (%) WB+** | **OR** |
| **Overall** | **7** | **4 (57.1)** |  | **494** | **37 (7.5)** |  | **221** | **22 (10.0)** |  | **155** | **18 (11.6)** |  | **23** | **2 ( 8.7)** |  | **603** | **31 (5.1)** |  | **96** | **9 (9.4)** |  | **507** | **22 (4.3)** |  |
| **Age** |  | **P=.72** |  |  | **P=.71** |  |  | **P=.77** |  |  | **P=.82** |  |  | **P=.97** |  |  | **P=.16** |  |  | **P=.32** |  |  | **P=.13** |  |
| **1-5 mos** | 1 | 1 (100) | ---- | 269 | 20 (7.4) | ---- | 116 | 11 (9.5) | ---- | 83 | 9 (10.8) | ---- | 9 | 1 (11.1) | ---- | 274 | 11 (4.0) | ---- | 27 | 1 (3.7) | ---- | 247 | 10 (4.0) | ---- |
| **6-11 mos** | 2 | 2 (100) | 1.68 | 112 | 11 (9.8) | 1.38 | 50 | 7 (14.0) | 1.58 | 37 | 6 (16.2) | 1.62 | 7 | 1 (14.3) | 1.31 | 154 | 6 (3.9) | 1 | 32 | 2 (6.3) | 1.45 | 122 | 4 (3.3) | 0.86 |
| **12-23 mos** | 3 | 1 (33.3) | 0.2 | 67 | 4 (6.0) | 0.86 | 38 | 3 (7.9) | 0.9 | 23 | 2 (8.7) | 0.91 | 5 | 0 (0) | 0.52 | 106 | 10 (9.4) | 2.49 | 18 | 2 (11.1) | 2.68 | 88 | 8 (9.1) | 2.39 |
| **24-59 mos** | 1 | 0 (0) | 0.11 | 46 | 2 (4.3) | 0.68 | 17 | 1 (5.9) | 0.83 | 12 | 1 (8.3) | 1.02 | 2 | 0 (0) | 1.13 | 69 | 4 (5.8) | 1.57 | 19 | 4 (21.1) | 5.13 | 50 | 0 (0) | 0.22 |
| **Gender** |  | **P=.40** |  |  | **P=.60** |  |  | **P=.48** |  |  | **P=.18** |  |  | **P=.67** |  |  | **P=.10** |  |  | **P=.85** |  |  | **P=.04** |  |
| **Female** | 4 | 3 (75.0) | 3.89 | 220 | 18 (8.2) | 1.2 | 95 | 11 (11.6) | 1.37 | 63 | 10 (15.9) | 1.95 | 17 | 2 (11.8) | 2.1 | 300 | 20 (6.7) | 1.86 | 46 | 4 (8.7) | 0.88 | 254 | 16 (6.3) | 2.63 |
| **Male** | 3 | 1 (33.3) | ---- | 274 | 19 (6.9) | ---- | 126 | 11 (8.7) | ---- | 92 | 8 (8.7) | ---- | 6 | 0 (0) | ---- | 303 | 11 (3.6) | ---- | 50 | 5 (10.0) | ---- | 253 | 6 (2.4) | ---- |
| **HIV infected** |  | **P=.84** |  |  | **P=.16** |  |  | **P=.81** |  |  | **P=.82** |  |  | **P=.16** |  |  | **P=.04** |  |  | **P=.10** |  |  | **P>.99** |  |
| **Yes** | 5 | 3 (60.0) | 1.4 | 82 | 9 (11.0) | 1.74 | 48 | 5 (10.4) | 1.13 | 41 | 5 (12.2) | 1.13 | 3 | 1 (33.3) | 7.8 | 70 | 7 (10.0) | 2.45 | 38 | 6 (15.8) | 3.17 | 32 | 1 (3.1) | 1 |
| **No** | 2 | 1 (50.0) | ---- | 412 | 28 (6.8) | ---- | 173 | 17 (9.8) | ---- | 114 | 13 (11.4) | ---- | 20 | 1 (5.0) | ---- | 531 | 24 (4.5) | ---- | 58 | 3 ( 5.2) | ---- | 473 | 21 (4.4) | ---- |
| **Very severe pneumonia** |  | **P=.65** |  |  | **P=.08** |  |  | **P=.17** |  |  | **P=.16** |  |  | **P=.58** |  |  | **--** |  |  | **--** |  |  | **--** |  |
| **Yes** | 1 | 1 (100) | 3.11 | 162 | 17 (10.5) | 1.83 | 81 | 11 (13.6) | 1.84 | 62 | 10 (16.1) | 2.01 | 7 | 0 (0) | 0.39 | ---- | N/A | ---- | ---- | N/A | ---- | ---- | N/A | ---- |
| **No** | 6 | 3 (50.0) | ---- | 332 | 20 (6.0) | ---- | 140 | 11 (7.9) | ---- | 93 | 8 (8.6) | ---- | 16 | 2 (12.5) | ---- | ---- | N/A | ---- | ---- | N/A | ---- | ---- | N/A | ---- |
| **Prior antibiotic use^e^** |  | **P=.55** |  |  | **P=.83** |  |  | **P=.93** |  |  | **P=.92** |  |  | **P=.77** |  |  | **P=.94** |  |  | **P=.97** |  |  | **P=.78** |  |
| **Yes** | 3 | 1 (33.3) | 0.36 | 450 | 33 (7.3) | 0.88 | 203 | 20 (9.9) | 1.08 | 140 | 16 (11.4) | 1.1 | 19 | 1 (5.3) | 0.57 | 26 | 1 (3.8) | 0.94 | 3 | 0 (0) | 1.08 | 23 | 1 (4.3) | 1.29 |
| **No** | 3 | 2 (66.7) | ---- | 41 | 3 (7.3) | ---- | 15 | 1 (6.7) | ---- | 13 | 1 (7.7) | ---- | 3 | 0 (0) | ---- | 515 | 30 (5.8) | ---- | 80 | 9 (11.3) | ---- | 435 | 21 (4.8) | ---- |
| **NP culture positive for Pneumococcus** |  | **P=.84** |  |  | **P=.08** |  |  | **P=.37** |  |  | **P=.62** |  |  | **P=.64** |  |  | **P=.93** |  |  | **P=.65** |  |  | **P=.90** |  |
| **Yes** | 5 | 3 (60.0) | 1.4 | 187 | 19 (10.2) | 1.81 | 91 | 11 (12.1) | 1.48 | 61 | 8 (13.1) | 1.28 | 6 | 0 (0) | 0.45 | 382 | 20 (5.2) | 1.04 | 56 | 6 (10.7) | 1.38 | 326 | 14 (4.3) | 0.95 |
| **No** | 2 | 1 (50.0) | ---- | 307 | 18 ( 5.9) | ---- | 130 | 11 (8.5) | ---- | 94 | 10 (10.6) | ---- | 16 | 2 (12.5) | ---- | 221 | 11 (5.0) | ---- | 40 | 3 (7.5) | ---- | 181 | 8 (4.4) | ---- |
| **Pneumococcus colonized (culture or PCR positive)** |  | **--** |  |  | **P=.25** |  |  | **P=.53** |  |  | **P=.77** |  |  | **P=.86** |  |  | **P=.64** |  |  | **P=.31** |  |  | **P=.93** |  |
| **Yes** | 6 | 4 (66.7) | ---- | 375 | 32 (8.5) | 1.82 | 167 | 18 (10.8) | 1.47 | 115 | 14 (12.2) | 1.2 | 18 | 2 (11.1) | 1.36 | 505 | 26 (5.1) | 0.8 | 79 | 7 (8.9) | 0.43 | 426 | 19 (4.5) | 0.95 |
| **No** | 0 | 0 (0) | ---- | 90 | 4 (4.4) | ---- | 44 | 3 (6.8) | ---- | 32 | 3 (9.4) | ---- | 4 | 0 (0) | ---- | 84 | 5 (6.0) | ---- | 12 | 2 (16.7) | ---- | 72 | 3 (4.2) | ---- |
| **Pneumococcal NP/OP PCR density >6.9 log10 copies/mL** |  | **P=.25** |  |  | **P=.94** |  |  | **P=.46** |  |  | **P=.43** |  |  | **P=.69** |  |  | **P=.73** |  |  | **P=.06** |  |  | **P=.43** |  |
| **Yes** | 3 | 3 (100) | 11.7 | 41 | 3 (7.3) | 1.05 | 23 | 1 (4.3) | 0.52 | 20 | 1 (5.0) | 0.49 | 1 | 0 (0) | 2.6 | 36 | 2 (5.6) | 1.27 | 6 | 2 (33.3) | 5.94 | 30 | 0 (0) | 0.31 |
| **No** | 3 | 1 (33.3) | ---- | 406 | 32 (7.9) | ---- | 179 | 20 (11.2) | ---- | 120 | 16 (13.3) | ---- | 21 | 2 ( 9.5) | ---- | 508 | 27 (5.3) | ---- | 75 | 6 (8.0) | ---- | 433 | 21 (4.8) | ---- |
| **NP/OP PCR positive for any virus** |  | **--** |  |  | **P=.05** |  |  | **P=.21** |  |  | **P=.33** |  |  | **P=.69** |  |  | **P=.62** |  |  | **P=.69** |  |  | **P=.95** |  |
| **Yes** | 6 | 4 (66.7) | ---- | 382 | 26 (6.8) | 0.45 | 172 | 16 (9.3) | 0.51 | 118 | 13 (11.0) | 0.55 | 21 | 2 ( 9.5) | 0.38 | 427 | 24 (5.6) | 1.28 | 75 | 8 (10.7) | 1.89 | 352 | 16 (4.5) | 0.97 |
| **No** | 0 | 0 (0) | ---- | 66 | 9 (13.6) | ---- | 31 | 5 (16.1) | ---- | 23 | 4 (17.4) | ---- | 1 | 0 (0) | ---- | 120 | 5 (4.2) | ---- | 7 | 0 (0) | ---- | 113 | 5 (4.4) | ---- |
| **Hypoxemia^f^** |  | **P=.40** |  |  | **P=.04** |  |  | **P=.17** |  |  | **P=.17** |  |  | **P=.77** |  |  | **--** |  |  | **--** |  |  | **--** |  |
| **Yes** | 4 | 3 (75.0) | 3.89 | 199 | 21 (10.6) | 2.03 | 108 | 14 (13.0) | 1.89 | 78 | 12 (15.4) | 2.04 | 9 | 1 (11.1) | 1.47 | ---- | N/A | ---- | ---- | N/A | ---- | ---- | N/A | ---- |
| **No** | 3 | 1 (33.3) | ---- | 294 | 16 (5.4) | ---- | 112 | 8 (7.1) | ---- | 76 | 6 ( 7.9) | ---- | 13 | 1 ( 7.7) | ---- | ---- | N/A | ---- | ---- | N/A | ---- | ---- | N/A | ---- |
| **Died in hospital** |  | **P=.65** |  |  | **P=.71** |  |  | **P=.90** |  |  | **P=.76** |  |  | **P=.83** |  |  | **--** |  |  | **--** |  |  | **--** |  |
| **Yes** | 1 | 1 (100) | 3.11 | 86 | 7 (8.1) | 1.17 | 31 | 3 (9.7) | 1.08 | 24 | 3 (12.5) | 1.22 | 10 | 1 (10.0) | 1.32 | ---- | N/A | ---- | ---- | N/A | ---- | ---- | N/A | ---- |
| **No** | 6 | 3 (50.0) | ---- | 408 | 30 (7.4) | ---- | 190 | 19 (10.0) | ---- | 131 | 15 (11.5) | ---- | 13 | 1 ( 7.7) | ---- | ---- | N/A | ---- | ---- | N/A | ---- | ---- | N/A | ---- |
| **CXR+** |  | **P=.56** |  |  | **P=.051** |  |  | **--** |  |  | **--** |  |  | **P=.51** |  |  | **--** |  |  | **--** |  |  | **--** |  |
| **Yes** | 5 | 2 (40.0) | 0.24 | 221 | 22 (10.0) | 2.59 | 221 | 22 (10.0) | ---- | 155 | 18 (11.6) | ---- | 10 | 2 (20.0) | 3.23 | ---- | N/A | ---- | ---- | N/A | ---- | ---- | N/A | ---- |
| **No** | 1 | 1 (100) | ---- | 131 | 5 (3.8) | ---- | 0 | 0 (0) | ---- | 0 | 0 (0) | ---- | 5 | 0 (0) | ---- | ---- | N/A | ---- | ---- | N/A | ---- | ---- | N/A | ---- |
| **Alveolar consolidation on CXR** |  | **P=.55** |  |  | **P=.02** |  |  | **P=.26** |  |  | **--** |  |  | **P=.18** |  |  | **--** |  |  | **--** |  |  | **--** |  |
| **Yes** | 3 | 2 (66.7) | 2.78 | 155 | 18 (11.6) | 2.67 | 155 | 18 (11.6) | 1.87 | 155 | 18 (11.6) | ---- | 6 | 2 (33.3) | 10.6 | ---- | N/A | ---- | ---- | N/A | ---- | ---- | N/A | ---- |
| **No** | 3 | 1 (33.3) | ---- | 197 | 9 ( 4.6) | ---- | 66 | 4 (6.1) | ---- | 0 | 0 (0) | ---- | 9 | 0 (0) | ---- | ---- | N/A | ---- | ---- | N/A | ---- | ---- | N/A | ---- |
| **WBC >15mm^3^** |  | **P=.18** |  |  | **P=.71** |  |  | **P=.65** |  |  | **P=.76** |  |  | **P=.47** |  |  | **--** |  |  | **--** |  |  | **--** |  |
| **Yes** | 4 | 1 (25.0) | 0.06 | 187 | 13 (7.0) | 0.88 | 99 | 11 (11.1) | 1.23 | 74 | 8 (10.8) | 0.86 | 8 | 0 (0) | 0.29 | ---- | N/A | ---- | ---- | N/A | ---- | ---- | N/A | ---- |
| **No** | 3 | 3 (100) | ---- | 301 | 24 (8.0) | ---- | 119 | 11 (9.2) | ---- | 80 | 10 (12.5) | ---- | 14 | 2 (14.3) | ---- | ---- | N/A | ---- | ---- | N/A | ---- | ---- | N/A | ---- |
| **CRP >40mg/L** |  | **P=.56** |  |  | **P=.30** |  |  | **P=.92** |  |  | **P>0.99** |  |  | **--** |  |  | **--** |  |  | **--** |  |  | **--** |  |
| **Yes** | 5 | 3 (60.0) | 4.24 | 169 | 16 (9.5) | 1.43 | 102 | 10 (9.8) | 0.95 | 78 | 9 (11.5) | 1.01 | 12 | 0 (0) | ---- | ---- | N/A | ---- | ---- | N/A | ---- | ---- | N/A | ---- |
| **No** | 1 | 0 (0) | ---- | 292 | 20 (6.8) | ---- | 107 | 11 (10.3) | ---- | 70 | 8 (11.4) | ---- | 6 | 0 (0) | ---- | ---- | N/A | ---- | ---- | N/A | ---- | ---- | N/A | ---- |

N/A, data not collected for controls.

Abbreviations: MCPP, microbiologically confirmed pneumococcal pneumonia; WB, whole blood; PCV, pneumococcal conjugate vaccine; CXR, chest radiograph; CXR-AC, alveolar consolidation on CXR; WBC, white blood cells; CRP, C-reactive protein; HIV, human immunodeficiency virus; PCR, polymerase chain reaction; NP, nasopharyngeal; OP, oropharyngeal; RTI, respiratory tract illness.

^a^ MCPP defined as isolation of pneumococcus from blood culture, culture or PCR of lung aspirate or pleural fluid, or BinaxNOW antigen detection on pleural fluid.

^b^Non-confirmed cases defined as cases without isolation of bacteria from culture of blood, lung aspirate or pleural fluid, or PCR of lung aspirate or pleural fluid.

^c^ CXR+ defined as radiographic evidence of pneumonia (consolidation and/or other infiltrates).

^d^ Confirmed non-pneumococcal bacterial case was defined as a case with any non-pneumococcal bacterial pathogen detected by blood culture, by lung aspirate culture or PCR, or by pleural fluid culture or PCR.

^e^ Prior use of antibiotics defined as serum bioassay positive, antibiotic administration at the referral facility, or antibiotic administration prior to blood specimen collection at the study facility.

^f^ Hypoxemia was defined as <92% on room air (<90% at elevation, Zambia and South Africa) or a requirement for supplemental oxygen if a room air reading was not available.

**Supplementary Tables 2E Whole blood pneumococcal PCR positivity by PERCH case-control group and clinical characteristics**

| **South Africa** | **All MCPP cases^a^ N=5** | | | **Non-Confirmed cases^b^ N=885** | | | **Non-Confirmed CXR+^c^ cases N=500** | | | **Non-Confirmed CXR-AC cases N=290** | | | **Confirmed non-pneu bacterial case^d^ N=27** | | | **All controls N=963** | | | **RTI controls N=53** | | | **Non-RTI controls N=910** | | |
| --- | --- | --- | --- | --- | --- | --- | --- | --- | --- | --- | --- | --- | --- | --- | --- | --- | --- | --- | --- | --- | --- | --- | --- | --- |
|  | **N** | **n (%) WB+** | **OR** | **N** | **n (%) WB+** | **OR** | **N** | **n (%) WB+** | **OR** | **N** | **n (%) WB+** | **OR** | **N** | **n (%) WB+** | **OR** | **N** | **n (%) WB+** | **OR** | **N** | **n (%) WB+** | **OR** | **N** | **n (%) WB+** | **OR** |
| **Overall** | **5** | **4 (80.0)** |  | **885** | **66 (7.5)** |  | **500** | **44 (8.8)** |  | **290** | **30 (10.3)** |  | **27** | **1 (3.7)** |  | **963** | **98 (10.2)** |  | **53** | **8 (15.1)** |  | **910** | **90 (9.9)** |  |
| **Age** |  | **--** |  |  | **P=.19** |  |  | **P=.11** |  |  | **P=.11** |  |  | **P=.35** |  |  | **P=.33** |  |  | **P=.14** |  |  | **P=.20** |  |
| **1-5 mos** | 0 | 0 (0) | ---- | 441 | 26 (5.9) | ---- | 244 | 18 (7.4) | ---- | 143 | 11 (7.7) | ---- | 15 | 0 (0) | ---- | 365 | 31 (8.5) | ---- | 16 | 1 (6.3) | ---- | 349 | 30 (8.6) | ---- |
| **6-11 mos** | 2 | 1 (50.0) | ---- | 216 | 17 (7.9) | 1.38 | 131 | 11 (8.4) | 1.17 | 77 | 7 (9.1) | 1.23 | 8 | 0 (0) | 1.82 | 268 | 32 (11.9) | 1.46 | 19 | 4 (21.1) | 3 | 249 | 28 (11.2) | 1.35 |
| **12-23 mos** | 2 | 2 (100) | ---- | 152 | 14 (9.2) | 1.64 | 90 | 8 (8.9) | 1.26 | 49 | 7 (14.3) | 2.03 | 2 | 0 (0) | 6.2 | 168 | 21 (12.5) | 1.55 | 12 | 0 (0) | 0.41 | 156 | 21 (13.5) | 1.66 |
| **24-59 mos** | 1 | 1 (100) | ---- | 76 | 9 (11.8) | 2.21 | 35 | 7 (20.0) | 3.22 | 21 | 5 (23.8) | 3.84 | 2 | 1 (50.0) | 31 | 162 | 14 (8.6) | 1.04 | 6 | 3 (50.0) | 10.3 | 156 | 11 (7.1) | 0.83 |
| **Gender** |  | **P=.92** |  |  | **P=.65** |  |  | **P=.69** |  |  | **P=.69** |  |  | **P=.53** |  |  | **P=.09** |  |  | **P=.95** |  |  | **P=.08** |  |
| **Female** | 4 | 3 (75.0) | 0.76 | 413 | 29 (7.0) | 0.89 | 247 | 23 (9.3) | 1.13 | 155 | 15 (9.7) | 0.86 | 14 | 1 (7.1) | 3 | 501 | 59 (11.8) | 1.44 | 27 | 4 (14.8) | 0.96 | 474 | 55 (11.6) | 1.5 |
| **Male** | 1 | 1 (100) | ---- | 472 | 37 (7.8) | ---- | 253 | 21 (8.3) | ---- | 135 | 15 (11.1) | ---- | 13 | 0 (0) | ---- | 462 | 39 (8.4) | ---- | 26 | 4 (15.4) | ---- | 436 | 35 (8.0) | ---- |
| **HIV infected** |  | **P=.39** |  |  | **P=.08** |  |  | **P=.09** |  |  | **P=.20** |  |  | **P=.24** |  |  | **P=.62** |  |  | **P=.98** |  |  | **P=.65** |  |
| **Yes** | 2 | 1 (50.0) | 0.14 | 105 | 12 (11.4) | 1.78 | 82 | 11 (13.4) | 1.85 | 54 | 8 (14.8%) | 1.74 | 8 | 1 (12.5) | 7.8 | 136 | 12 (8.8) | 0.85 | 8 | 1 (12.5) | 1.03 | 128 | 11 (8.6) | 0.86 |
| **No** | 3 | 3 (100) | ---- | 780 | 54 (6.9) | ---- | 418 | 33 (7.9) | ---- | 236 | 22 ( 9.3%) | ---- | 19 | 0 (0) | ---- | 822 | 86 (10.5) | ---- | 45 | 7 (15.6) | ---- | 777 | 79 (10.2) | ---- |
| **PCV vaccinated^e^** |  | **--** |  |  | **P=.09** |  |  | **P=.04** |  |  | **P=.01** |  |  | **--** |  |  | **P=.10** |  |  | **P=.97** |  |  | **P=.14** |  |
| **Yes** | 5 | 4 (80.0) | ---- | 619 | 39 (6.3) | 0.62 | 352 | 25 (7.1) | 0.5 | 201 | 14 (7.0) | 0.32 | 17 | 0 (0) | ---- | 777 | 84 (10.8) | 1.91 | 47 | 8 (17.0) | 1.08 | 730 | 76 (10.4) | 1.8 |
| **No** | 0 | 0 (0) | ---- | 206 | 20 (9.7) | ---- | 114 | 15 (13.2) | ---- | 64 | 12 (18.8) | ---- | 8 | 0 (0) | ---- | 124 | 7 (5.6) | ---- | 2 | 0 (0) | ---- | 122 | 7 (5.7) | ---- |
| **Very severe pneumonia** |  | **P=.62** |  |  | **P=.17** |  |  | **P=.98** |  |  | **P=.72** |  |  | **P=.65** |  |  | **--** |  |  | **--** |  |  | **--** |  |
| **Yes** | 3 | 2 (66.7) | 0.33 | 283 | 26 (9.2) | 1.43 | 171 | 15 (8.8) | 1.01 | 108 | 12 (11.1) | 1.15 | 11 | 0 (0) | 0.45 | ---- | N/A | ---- | ---- | N/A | ---- | ---- | N/A | ---- |
| **No** | 2 | 2 (100) | ---- | 602 | 40 (6.6) | ---- | 329 | 29 (8.8) | ---- | 182 | 18 (9.9) | ---- | 16 | 1 (6.3) | ---- | ---- | N/A | ---- | ---- | N/A | ---- | ---- | N/A | ---- |
| **Prior antibiotic use^f^** |  | **P=.92** |  |  | **P=.72** |  |  | **P=.79** |  |  | **P=.75** |  |  | **P=.42** |  |  | **P=.51** |  |  | **P=.83** |  |  | **P=.64** |  |
| **Yes** | 4 | 3 (75.0) | 0.76 | 458 | 33 (7.2) | 0.91 | 279 | 26 (9.3) | 1.09 | 162 | 16 (9.9) | 0.88 | 15 | 0 (0) | 0.25 | 24 | 1 (4.2) | 0.56 | 3 | 0 (0) | 0.68 | 21 | 1 (4.8) | 0.67 |
| **No** | 1 | 1 (100) | ---- | 369 | 29 (7.9) | ---- | 188 | 16 (8.5) | ---- | 109 | 12 (11.0) | ---- | 12 | 1 (8.3) | ---- | 857 | 87 (10.2) | ---- | 42 | 7 (16.7) | ---- | 815 | 80 (9.8) | ---- |
| **NP culture positive for Pneumococcus** |  | **P=.91** |  |  | **P<.001** |  |  | **P=.004** |  |  | **P=.004** |  |  | **P=.33** |  |  | **P=.02** |  |  | **P=.93** |  |  | **P=.02** |  |
| **Yes** | 1 | 1 (100) | 1.34 | 387 | 42 (10.9) | 2.47 | 227 | 29 (12.8) | 2.64 | 135 | 22 (16.3) | 3.42 | 10 | 1 (10.0) | 5.53 | 602 | 72 (12.0) | 1.73 | 38 | 6 (15.8) | 1.08 | 564 | 66 (11.7) | 1.76 |
| **No** | 4 | 3 (75.0) | ---- | 494 | 23 (4.7) | ---- | 271 | 14 (5.2) | ---- | 154 | 8 (5.2) | ---- | 17 | 0 (0) | ---- | 361 | 26 (7.2) | ---- | 15 | 2 (13.3) | ---- | 346 | 24 (6.9) | ---- |
| **Pneumococcus colonized (culture or PCR positive)** |  | **--** |  |  | **P<.001** |  |  | **P=.005** |  |  | **P=.01** |  |  | **P=.53** |  |  | **P=.007** |  |  | **P=.27** |  |  | **P=.02** |  |
| **Yes** | 5 | 4 (80.0) | ---- | 609 | 60 (9.9) | 5.37 | 360 | 40 (11.1) | 4.89 | 209 | 29 (13.9) | 8.66 | 14 | 1 (7.1) | 3 | 699 | 83 (11.9) | 2.16 | 42 | 8 (19.0) | 5.66 | 657 | 75 (11.4) | 1.98 |
| **No** | 0 | 0 (0) | ---- | 273 | 5 (1.8) | ---- | 138 | 3 (2.2) | ---- | 80 | 1 (1.3) | ---- | 13 | 0 (0) | ---- | 262 | 15 (5.7) | ---- | 11 | 0 (0) | ---- | 251 | 15 (6.0) | ---- |
| **Pneumococcal NP/OP PCR density >6.9 log10 copies/mL** |  | **P=.62** |  |  | **P<.001** |  |  | **P<.001** |  |  | **P<.001** |  |  | **P=.20** |  |  | **P=.09** |  |  | **P=.72** |  |  | **P=.09** |  |
| **Yes** | 2 | 2 (100) | 3 | 100 | 18 (18.0) | 3.47 | 70 | 15 (21.4) | 3.92 | 52 | 13 (25.0) | 4.31 | 7 | 1 (14.3) | 9.46 | 94 | 14 (14.9) | 1.69 | 6 | 1 (16.7) | 1.47 | 88 | 13 (14.8) | 1.73 |
| **No** | 3 | 2 (66.7) | ---- | 781 | 47 (6.0) | ---- | 428 | 28 (6.5) | ---- | 237 | 17 (7.2) | ---- | 20 | 0 (0) | ---- | 864 | 83 (9.6) | ---- | 47 | 7 (14.9) | ---- | 817 | 76 (9.3) | ---- |
| **NP/OP PCR positive for any virus** |  | **P=.62** |  |  | **P=.98** |  |  | **P=.21** |  |  | **P=.14** |  |  | **P=.89** |  |  | **P=.33** |  |  | **P=.30** |  |  | **P=.56** |  |
| **Yes** | 3 | 2 (66.7) | 0.33 | 755 | 56 (7.4) | 1.01 | 443 | 36 (8.1) | 0.58 | 261 | 25 (9.6) | 0.46 | 22 | 1 (4.5) | 0.77 | 709 | 76 (10.7) | 1.28 | 43 | 8 (18.6) | 5.03 | 666 | 68 (10.2) | 1.16 |
| **No** | 2 | 2 (100) | ---- | 127 | 9 (7.1) | ---- | 55 | 7 (12.7) | ---- | 28 | 5 (17.9) | ---- | 5 | 0 (0) | ---- | 249 | 21 (8.4) | ---- | 10 | 0 (0) | ---- | 239 | 21 (8.8) | ---- |
| **Hypoxemia^g^** |  | **P=.62** |  |  | **P=.97** |  |  | **P=.49** |  |  | **P=.51** |  |  | **P=.16** |  |  | **--** |  |  | **--** |  |  | **--** |  |
| **Yes** | 2 | 2 (100) | 3 | 666 | 50 (7.5) | 0.99 | 382 | 32 (8.4) | 0.78 | 224 | 22 (9.8) | 0.75 | 21 | 0 (0) | 0.09 | ---- | N/A | ---- | ---- | N/A | ---- | ---- | N/A | ---- |
| **No** | 3 | 2 (66.7) | ---- | 215 | 16 (7.4) | ---- | 117 | 12 (10.3) | ---- | 65 | 8 (12.3) | ---- | 6 | 1 (16.7) | ---- | ---- | N/A | ---- | ---- | N/A | ---- | ---- | N/A | ---- |
| **Died in hospital** |  | **--** |  |  | **P>.99** |  |  | **P=.86** |  |  | **P=.72** |  |  | **P=.98** |  |  | **--** |  |  | **--** |  |  | **--** |  |
| **Yes** | 0 | 0 (0) | ---- | 32 | 2 (6.3) | 1 | 24 | 2 (8.3) | 1.13 | 18 | 2 (11.1) | 1.29 | 6 | 0 (0) | 1.05 | ---- | N/A | ---- | ---- | N/A | ---- | ---- | N/A | ---- |
| **No** | 5 | 4 (80.0) | ---- | 852 | 64 (7.5) | ---- | 475 | 42 (8.8) | ---- | 271 | 28 (10.3) | ---- | 21 | 1 (4.8) | ---- | ---- | N/A | ---- | ---- | N/A | ---- | ---- | N/A | ---- |
| **CXR+** |  | **--** |  |  | **P=.13** |  |  | **--** |  |  | **--** |  |  | **P=.90** |  |  | **--** |  |  | **--** |  |  | **--** |  |
| **Yes** | 5 | 4 (80.0) | ---- | 500 | 44 (8.8) | 1.6 | 500 | 44 (8.8) | ---- | 290 | 30 (10.3) | ---- | 16 | 1 (6.3) | 1.26 | ---- | N/A | ---- | ---- | N/A | ---- | ---- | N/A | ---- |
| **No** | 0 | 0 (0) | ---- | 252 | 14 (5.6) | ---- | 0 | 0 (0) | ---- | 0 | 0 (0) | ---- | 6 | 0 (0) | ---- | ---- | N/A | ---- | ---- | N/A | ---- | ---- | N/A | ---- |
| **Alveolar consolidation on CXR** |  | **--** |  |  | **P=.03** |  |  | **P=.17** |  |  | **--** |  |  | **P=.57** |  |  | **--** |  |  | **--** |  |  | **--** |  |
| **Yes** | 5 | 4 (80.0) | ---- | 290 | 30 (10.3) | 1.78 | 290 | 30 (10.3) | 1.59 | 290 | 30 (10.3) | ---- | 12 | 1 (8.3) | 2.74 | ---- | N/A | ---- | ---- | N/A | ---- | ---- | N/A | ---- |
| **No** | 0 | 0 (0) | ---- | 462 | 28 (6.1) | ---- | 210 | 14 (6.7) | ---- | 0 | 0 (0) | ---- | 10 | 0 (0) | ---- | ---- | N/A | ---- | ---- | N/A | ---- | ---- | N/A | ---- |
| **WBC >15mm^3^** |  | **P=.92** |  |  | **P=.50** |  |  | **P=.85** |  |  | **P=.97** |  |  | **P=.50** |  |  | **--** |  |  | **--** |  |  | **--** |  |
| **Yes** | 4 | 3 (75.0) | 0.76 | 370 | 25 (6.8) | 0.84 | 220 | 20 (9.1) | 1.06 | 136 | 14 (10.3) | 0.99 | 13 | 0 (0) | 0.31 | ---- | N/A | ---- | ---- | N/A | ---- | ---- | N/A | ---- |
| **No** | 1 | 1 (100) | ---- | 512 | 41 (8.0) | ---- | 278 | 24 (8.6) | ---- | 153 | 16 (10.5) | ---- | 13 | 1 (7.7) | ---- | ---- | N/A | ---- | ---- | N/A | ---- | ---- | N/A | ---- |
| **CRP >40mg/L** |  | **--** |  |  | **P<.001** |  |  | **P<.001** |  |  | **P=.002** |  |  | **P=.59** |  |  | **--** |  |  | **--** |  |  | **--** |  |
| **Yes** | 5 | 4 (80.0) | ---- | 219 | 31 (14.2) | 3.01 | 154 | 24 (15.6) | 2.93 | 110 | 20 (18.2) | 3.57 | 15 | 1 (6.7) | 2.59 | ---- | N/A | ---- | ---- | N/A | ---- | ---- | N/A | ---- |
| **No** | 0 | 0 (0) | ---- | 655 | 34 (5.2) | ---- | 339 | 20 (5.9) | ---- | 175 | 10 (5.7) | ---- | 12 | 0 (0) | ---- | ---- | N/A | ---- | ---- | N/A | ---- | ---- | N/A | ---- |

N/A, data not collected for controls.

Abbreviations: MCPP, microbiologically confirmed pneumococcal pneumonia; WB, whole blood; PCV, pneumococcal conjugate vaccine; CXR, chest radiograph; CXR-AC, alveolar consolidation on CXR; WBC, white blood cells; CRP, C-reactive protein; HIV, human immunodeficiency virus; PCR, polymerase chain reaction; NP, nasopharyngeal; OP, oropharyngeal; RTI, respiratory tract illness.

^a^ MCPP defined as isolation of pneumococcus from blood culture, culture or PCR of lung aspirate or pleural fluid, or BinaxNOW antigen detection on pleural fluid.

^b^Non-confirmed cases defined as cases without isolation of bacteria from culture of blood, lung aspirate or pleural fluid, or PCR of lung aspirate or pleural fluid.

^c^ CXR+ defined as radiographic evidence of pneumonia (consolidation and/or other infiltrates).

^d^ Confirmed non-pneumococcal bacterial case was defined as a case with any non-pneumococcal bacterial pathogen detected by blood culture, by lung aspirate culture or PCR, or by pleural fluid culture or PCR.

^e^ PCV vaccinated defined as at least 1 dose.

^f^ Prior use of antibiotics defined as serum bioassay positive, antibiotic administration at the referral facility, or antibiotic administration prior to blood specimen collection at the study facility.

^g^ Hypoxemia was defined as <92% on room air (<90% at elevation, Zambia and South Africa) or a requirement for supplemental oxygen if a room air reading was not available.

**Supplementary Tables 2F Whole blood pneumococcal PCR positivity by PERCH case-control group and clinical characteristics**

| **Thailand** | **Non-Confirmed cases^a^  N=218** | | | **All controls N=622** | | | **RTI controls N=244** | | | **Non-RTI controls N=378** | | |
| --- | --- | --- | --- | --- | --- | --- | --- | --- | --- | --- | --- | --- |
|  | **N** | **n (%) WB+** | **OR** | **N** | **n (%) WB+** | **OR** | **N** | **n (%) WB+** | **OR** | **N** | **n (%) WB+** | **OR** |
| **Overall** | **218** | **3 (1.4)** |  | **622** | **5 (0.8)** |  | **244** | **4 (1.6)** |  | **378** | **1 (0.3)** |  |
| **Age** |  | **P=.59** |  |  | **P=.82** |  |  | **P=.96** |  |  | **P=.93** |  |
| **1-5 mos** | 35 | 1 (2.9) | ---- | 76 | 0 (0) | ---- | 21 | 0 (0) | ---- | 55 | 0 (0) | ---- |
| **6-11 mos** | 50 | 0 (0) | 0.23 | 144 | 1 (0.7) | 1.6 | 61 | 1 (1.6) | 1.07 | 83 | 0 (0) | 0.66 |
| **12-23 mos** | 68 | 0 (0) | 0.17 | 207 | 3 (1.4) | 2.62 | 80 | 2 (2.5) | 1.37 | 127 | 1 (0.8) | 1.32 |
| **24-59 mos** | 65 | 2 (3.1) | 0.91 | 195 | 1 (0.5) | 1.18 | 82 | 1 (1.2) | 0.79 | 113 | 0 (0) | 0.49 |
| **Gender** |  | **P=.35** |  |  | **P=.21** |  |  | **P=.36** |  |  | **P=.47** |  |
| **Female** | 85 | 2 (2.4) | 2.64 | 300 | 4 (1.3) | 3.25 | 120 | 3 (2.5) | 2.45 | 180 | 1 (0.6) | 3.32 |
| **Male** | 133 | 1 (0.8) | ---- | 322 | 1 (0.3) | ---- | 124 | 1 (0.8) | ---- | 198 | 0 (0) | ---- |
| **HIV infected** |  | **--** |  |  | **--** |  |  | **--** |  |  | **--** |  |
|  | 0 | 0 (0) | ---- | 0 | 0 (0) | ---- | 0 | 0 (0) | ---- | 0 | 0 (0) | ---- |
| **No** | 212 | 3 (1.4) | ---- | 619 | 5 (0.8) | ---- | 242 | 4 (1.7) | ---- | 377 | 1 (0.3) | ---- |
| **Very severe pneumonia** |  | **P=.51** |  |  | **--** |  |  | **--** |  |  | **--** |  |
| **Yes** | 50 | 1 (2.0) | 2.02 | ---- | N/A | ---- | ---- | N/A | ---- | ---- | N/A | ---- |
| **No** | 168 | 2 (1.2) |  | ---- | N/A | ---- | ---- | N/A | ---- | ---- | N/A | ---- |
| **Prior antibiotic use^b^** |  | **P=.46** |  |  | **P=.33** |  |  | **P=.58** |  |  | **P=.07** |  |
| **Yes** | 66 | 0 (0) | 0.32 | 10 | 0 (0) | 4.75 | 9 | 0 (0) | 2.42 | 1 | 0 (0) | 77.9 |
| **No** | 152 | 3 (2.0) | ---- | 553 | 5 ( 0.9) | ---- | 210 | 4 (1.9) | ---- | 343 | 1 (0.3) | ---- |
| **NP culture positive for Pneumococcus** |  | **P=.90** |  |  | **P=.31** |  |  | **P=.75** |  |  | **P=.46** |  |
| **Yes** | 88 | 1 (1.1) | 0.88 | 332 | 4 ( 1.2) | 2.64 | 154 | 3 (1.9) | 1.38 | 178 | 1 (0.6) | 3.39 |
| **No** | 130 | 2 (1.5) | ---- | 290 | 1 ( 0.3) | ---- | 90 | 1 (1.1) | ---- | 200 | 0 (0) | ---- |
| **Pneumococcus colonized (culture or PCR positive)** |  | **P=.83** |  |  | **P=.62** |  |  | **P=.93** |  |  | **P=.69** |  |
| **Yes** | 124 | 2 (1.6) | 1.26 | 405 | 4 (1.0) | 1.6 | 175 | 3 (1.7) | 0.91 | 230 | 1 (0.4) | 1.93 |
| **No** | 93 | 1 (1.1) | ---- | 215 | 1 (0.5) | ---- | 68 | 1 (1.5) | ---- | 147 | 0 (0) | ---- |
| **Pneumococcal NP/OP PCR density >6.9 log10 copies/mL** |  | **P=.24** |  |  | **P=.25** |  |  | **P=.45** |  |  | **P=.07** |  |
| **Yes** | 3 | 0 (0) | 8.59 | 8 | 0 (0) | 6.44 | 7 | 0 (0) | 3.4 | 1 | 0 (0) | 83.8 |
| **No** | 213 | 3 (1.4) | ---- | 606 | 5 (0.8) | ---- | 233 | 4 (1.7) | ---- | 373 | 1 (0.3) | ---- |
| **NP/OP PCR positive for any virus** |  | **P=.04** |  |  | **P=.15** |  |  | **P=.06** |  |  | **P=.84** |  |
| **Yes** | 181 | 1 (0.6) | 0.12 | 507 | 3 (0.6) | 0.3 | 203 | 2 (1.0) | 0.18 | 304 | 1 (0.3) | 0.72 |
| **No** | 37 | 2 (5.4) | ---- | 109 | 2 (1.8) | ---- | 37 | 2 (5.4) | ---- | 72 | 0 (0) | ---- |
| **Hypoxemia^c^** |  | **P=.60** |  |  | **--** |  |  | **--** |  |  | **--** |  |
| **Yes** | 52 | 0 (0) | 0.44 | 0 | 0 (0) | ---- | 0 | 0 (0) | ---- | 0 | 0 (0) | ---- |
| **No** | 166 | 3 (1.8) | ---- | 0 | 0 (0) | ---- | 0 | 0 (0) | ---- | 0 | 0 (0) | ---- |
| **Died in hospital** |  | **P=.20** |  |  | **--** |  |  | **--** |  |  | **--** |  |
| **Yes** | 1 | 0 (0) | 20.6 | ---- | N/A | ---- | ---- | N/A | ---- | ---- | N/A | ---- |
| **No** | 216 | 3 (1.4) | ---- | ---- | N/A | ---- | ---- | N/A | ---- | ---- | N/A | ---- |
| **CXR+^d^** |  | **P=.19** |  |  | **--** |  |  | **--** |  |  | **--** |  |
| **Yes** | 96 | 0 (0) | 0.14 | ---- | N/A | ---- | ---- | N/A | ---- | ---- | N/A | ---- |
| **No** | 94 | 3 (3.2) | ---- | ---- | N/A | ---- | ---- | N/A | ---- | ---- | N/A | ---- |
| **Alveolar consolidation on CXR** |  | **P=.66** |  |  | **--** |  |  | **--** |  |  | **--** |  |
| **Yes** | 41 | 0 (0) | 0.5 | ---- | N/A | ---- | ---- | N/A | ---- | ---- | N/A | ---- |
| **No** | 149 | 3 (2.0) | ---- | ---- | N/A | ---- | ---- | N/A | ---- | ---- | N/A | ---- |
| **WBC >15mm^3^** |  | **P=.16** |  |  | **--** |  |  | **--** |  |  | **--** |  |
| **Yes** | 98 | 3 (3.1) | 8.76 | ---- | N/A | ---- | ---- | N/A | ---- | ---- | N/A | ---- |
| **No** | 119 | 0 (0) | ---- | ---- | N/A | ---- | ---- | N/A | ---- | ---- | N/A | ---- |
| **CRP >40mg/L** |  | **P=.31** |  |  | **--** |  |  | **--** |  |  | **--** |  |
| **Yes** | 34 | 1 (2.9) | 2.93 | ---- | N/A | ---- | ---- | N/A | ---- | ---- | N/A | ---- |
| **No** | 165 | 2 (1.2) | ---- | ---- | N/A | ---- | ---- | N/A | ---- | ---- | N/A | ---- |

N/A, data not collected for controls.

Abbreviations: MCPP, microbiologically confirmed pneumococcal pneumonia; WB, whole blood; PCV, pneumococcal conjugate vaccine; CXR, chest radiograph; CXR-AC, alveolar consolidation on CXR; WBC, white blood cells; CRP, C-reactive protein; HIV, human immunodeficiency virus; PCR, polymerase chain reaction; NP, nasopharyngeal; OP, oropharyngeal; RTI, respiratory tract illness.

Restricted to case groups with WB pneumococcal PCR positive children.

^a^Non-confirmed cases defined as cases without isolation of bacteria from culture of blood, lung aspirate or pleural fluid, or PCR of lung aspirate or pleural fluid.

^b^ Prior use of antibiotics defined as serum bioassay positive, antibiotic administration at the referral facility, or antibiotic administration prior to blood specimen collection at the study facility.

^c^ Hypoxemia was defined as <92% on room air (<90% at elevation, Zambia and South Africa) or a requirement for supplemental oxygen if a room air reading was not available.

^d^ CXR+ defined as radiographic evidence of pneumonia (consolidation and/or other infiltrates).

**Supplementary Tables 2G Whole blood pneumococcal PCR positivity by PERCH case-control group and clinical characteristics**

| **Bangladesh** | **Non-Confirmed cases^a^ N=490** | | | **Non-Confirmed CXR+^b^ cases N=206** | | | **Non-Confirmed CXR-AC cases N=56** | | | **All controls N=725** | | | **RTI controls N=153** | | | **Non-RTI controls N=572** | | |
| --- | --- | --- | --- | --- | --- | --- | --- | --- | --- | --- | --- | --- | --- | --- | --- | --- | --- | --- |
|  | **N** | **n (%) WB+** | **OR** | **N** | **n (%) WB+** | **OR** | **N** | **n (%) WB+** | **OR** | **N** | **n (%) WB+** | **OR** | **N** | **n (%) WB+** | **OR** | **N** | **n (%) WB+** | **OR** |
| **Overall** | **490** | **5 (1.0)** |  | **206** | **1 (0.5)** |  | **56** | **1 (1.8)** |  | **725** | **6 (0.8)** |  | **153** | **4 (2.6)** |  | **572** | **2 (0.3)** |  |
| **Age** |  | **P=.82** |  |  | **P=.97** |  |  | **P=.91** |  |  | **P=.44** |  |  | **P=.43** |  |  | **P=.78** |  |
| **1-5 mos** | 121 | 1 (0.8) | ---- | 45 | 0 (0) | ---- | 15 | 0 (0) | ---- | 203 | 0 (0) | ---- | 56 | 0 (0) | ---- | 147 | 0 (0) | ---- |
| **6-11 mos** | 113 | 1 (0.9) | 1.07 | 48 | 0 (0) | 0.94 | 11 | 0 (0) | 1.35 | 155 | 3 (1.9) | 9.34 | 31 | 2 (6.5) | 9.58 | 124 | 1 (0.8) | 3.58 |
| **12-23 mos** | 168 | 3 (1.8) | 1.7 | 76 | 1 (1.3) | 1.81 | 17 | 1 (5.9) | 2.82 | 188 | 2 (1.1) | 5.46 | 38 | 2 (5.3) | 7.74 | 150 | 0 (0) | 0.98 |
| **24-59 mos** | 88 | 0 (0) | 0.45 | 37 | 0 (0) | 1.21 | 13 | 0 (0) | 1.15 | 179 | 1 (0.6) | 3.42 | 28 | 0 (0) | 1.98 | 151 | 1 (0.7) | 2.94 |
| **Gender** |  | **P=.77** |  |  | **P=.35** |  |  | **P=.45** |  |  | **P=.38** |  |  | **P=.97** |  |  | **P=.26** |  |
| **Female** | 176 | 2 (1.1) | 1.28 | 81 | 1 (1.2) | 4.68 | 26 | 1 (3.8) | 3.59 | 381 | 2 (0.5) | 0.5 | 78 | 2 (2.6) | 0.96 | 303 | 0 (0) | 0.18 |
| **Male** | 314 | 3 (1.0) | ---- | 125 | 0 (0) | ---- | 30 | 0 (0) | ---- | 344 | 4 (1.2) | ---- | 75 | 2 (2.7) | ---- | 269 | 2 (0.7) | ---- |
| **HIV infected** |  | **--** |  |  | **--** |  |  | **--** |  |  | **--** |  |  | **--** |  |  | **--** |  |
|  | 0 | 0 (0) | ---- | 0 | 0 (0) | ---- | 0 | 0 (0) | ---- | 0 | 0 (0) | ---- | 0 | 0 (0) | ---- | 0 | 0 (0) | ---- |
| **No** | 490 | 5 (1.0) | ---- | 206 | 1 (0.5) | ---- | 56 | 1 (1.8) | ---- | 725 | 6 (0.8) | ---- | 153 | 4 (2.6) | ---- | 572 | 2 (0.3) | ---- |
| **Very severe pneumonia** |  | **P=.26** |  |  | **P=.06** |  |  | **P=.1** |  |  | **--** |  |  | **--** |  |  | **--** |  |
| **Yes** | 50 | 1 (2.0) | 2.94 | 25 | 1 (4.0) | 22.2 | 9 | 1 (11.1) | 16.8 | ---- | N/A | ---- | ---- | N/A | ---- | ---- | N/A | ---- |
| **No** | 440 | 4 (0.9) | ---- | 181 | 0 (0) | ---- | 47 | 0 (0) | ---- | ---- | N/A | ---- | ---- | N/A | ---- | ---- | N/A | ---- |
| **Prior antibiotic use^c^** |  | **P=.37** |  |  | **P=.17** |  |  | **P=.22** |  |  | **P=.30** |  |  | **P=.48** |  |  | **P=.10** |  |
| **Yes** | 123 | 2 (1.6) | 2.13 | 50 | 1 (2.0) | 9.42 | 16 | 1 (6.3) | 7.84 | 10 | 0 (0) | 5.1 | 4 | 0 (0) | 3.42 | 6 | 0 (0) | 17.2 |
| **No** | 364 | 3 (0.8) | ---- | 155 | 0 (0) | ---- | 40 | 0 (0) | ---- | 702 | 6 (0.9) | ---- | 142 | 4 (2.8) | ---- | 560 | 2 (0.4) | ---- |
| **NP culture positive for Pneumococcus** |  | **P=.93** |  |  | **P=.69** |  |  | **P=.77** |  |  | **P=.42** |  |  | **P=.53** |  |  | **P=.90** |  |
| **Yes** | 293 | 3 (1.0) | 0.93 | 125 | 1 (0.8) | 1.94 | 36 | 1 (2.8) | 1.65 | 581 | 6 (1.0) | 3.26 | 120 | 4 (3.3) | 2.59 | 461 | 2 (0.4) | 1.21 |
| **No** | 195 | 2 (1.0) | ---- | 80 | 0 (0) | ---- | 19 | 0 (0) | ---- | 144 | 0 (0) | ---- | 33 | 0 (0) | ---- | 111 | 0 (0) | ---- |
| **Pneumococcus colonized (culture or PCR positive)** |  | **P=.46** |  |  | **P=.88** |  |  | **P=.88** |  |  | **P=.60** |  |  | **P=.74** |  |  | **P=.90** |  |
| **Yes** | 352 | 3 (0.9) | 0.54 | 144 | 1 (0.7) | 1.29 | 39 | 1 (2.6) | 1.29 | 621 | 6 (1.0) | 2.19 | 130 | 4 (3.1) | 1.67 | 491 | 2 (0.4) | 0.82 |
| **No** | 136 | 2 (1.5) | ---- | 61 | 0 (0) | ---- | 16 | 0 (0) | ---- | 103 | 0 (0) | ---- | 23 | 0 (0) | ---- | 80 | 0 (0) | ---- |
| **Pneumococcal NP/OP PCR density >6.9 log10 copies/mL** |  | **P=.06** |  |  | **P=.08** |  |  | **P=.09** |  |  | **P=.74** |  |  | **P=.85** |  |  | **P=.74** |  |
| **Yes** | 63 | 2 (3.2) | 4.93 | 30 | 1 (3.3) | 17.9 | 8 | 1 (12.5) | 19.4 | 79 | 0 (0) | 0.62 | 19 | 0 (0) | 0.74 | 60 | 0 (0) | 1.68 |
| **No** | 427 | 3 (0.7) | ---- | 176 | 0 (0) | ---- | 48 | 0 (0) | ---- | 643 | 6 (0.9) | ---- | 134 | 4 (3.0) | ---- | 509 | 2 ( 0.4) | ---- |
| **NP/OP PCR positive for any virus** |  | **P=.72** |  |  | **P=.38** |  |  | **P=.47** |  |  | **P=.70** |  |  | **P=.85** |  |  | **P=.86** |  |
| **Yes** | 466 | 5 (1.1) | 0.58 | 192 | 1 (0.5) | 0.23 | 52 | 1 (1.9) | 0.26 | 637 | 6 (0.9) | 1.76 | 142 | 4 (2.8) | 0.75 | 495 | 2 (0.4) | 0.75 |
| **No** | 24 | 0 (0) |  | 14 | 0 (0) |  | 4 | 0 (0) |  | 85 | 0 (0) |  | 11 | 0 (0) |  | 74 | 0 (0) |  |
| **Hypoxemia^d^** |  | **P>.99** |  |  | **P=.49** |  |  | **P=.34** |  |  | **--** |  |  | **--** |  |  | **--** |  |
| **Yes** | 40 | 0 (0) | 1 | 19 | 0 (0) | 3.19 | 2 | 0 (0) | 7.13 | ---- | N/A | ---- | ---- | N/A | ---- | ---- | N/A | ---- |
| **No** | 450 | 5 (1.1) | ---- | 187 | 1 (0.5) | ---- | 54 | 1 (1.9) | ---- | ---- | N/A | ---- | ---- | N/A | ---- | ---- | N/A | ---- |
| **Died in hospital** |  | **P=.14** |  |  | **--** |  |  | **--** |  |  | **--** |  |  | **--** |  |  | **--** |  |
| **Yes** | 2 | 0 (0) | 17.6 | 0 | 0 (0) | ---- | 0 | 0 (0) | ---- | ---- | N/A | ---- | ---- | N/A | ---- | ---- | N/A | ---- |
| **No** | 488 | 5 (1.0) | ---- | 206 | 1 (0.5) | ---- | 56 | 1 (1.8) | ---- | ---- | N/A | ---- | ---- | N/A | ---- | ---- | N/A | ---- |
| **CXR+** |  | **P=.30** |  |  | **--** |  |  | **--** |  |  | **--** |  |  | **--** |  |  | **--** |  |
| **Yes** | 206 | 1 (0.5) | 0.38 | 206 | 1 (0.5) | ---- | 56 | 1 (1.8) | ---- | ---- | N/A | ---- | ---- | N/A | ---- | ---- | N/A | ---- |
| **No** | 235 | 4 (1.7) | ---- | 0 | 0 (0) | ---- | 0 | 0 (0) | ---- | ---- | N/A | ---- | ---- | N/A | ---- | ---- | N/A | ---- |
| **Alveolar consolidation on CXR** |  | **P=.39** |  |  | **P=.20** |  |  | **--** |  |  | **--** |  |  | **--** |  |  | **--** |  |
| **Yes** | 56 | 1 (1.8) | 2.29 | 56 | 1 (1.8) | 8.13 | 56 | 1 (1.8) | ---- | ---- | N/A | ---- | ---- | N/A | ---- | ---- | N/A | ---- |
| **No** | 385 | 4 (1.0) | ---- | 150 | 0 (0) | ---- | 0 | 0 (0) | ---- | ---- | N/A | ---- | ---- | N/A | ---- | ---- | N/A | ---- |
| **WBC >15mm^3^** |  | **P=.51** |  |  | **P=.53** |  |  | **P=.63** |  |  | **--** |  |  | **--** |  |  | **--** |  |
| **Yes** | 215 | 3 (1.4) | 1.74 | 97 | 0 (0) | 0.36 | 23 | 0 (0) | 0.45 | ---- | N/A | ---- | ---- | N/A | ---- | ---- | N/A | ---- |
| **No** | 266 | 2 (0.8) | ---- | 105 | 1 (1.0) | ---- | 32 | 1 (3.1) | ---- | ---- | N/A | ---- | ---- | N/A | ---- | ---- | N/A | ---- |
| **CRP >40mg/L** |  | **P=.15** |  |  | **P=.06** |  |  | **P=.14** |  |  | **--** |  |  | **--** |  |  | **--** |  |
| **Yes** | 44 | 1 (2.3) | 4.15 | 23 | 1 (4.3) | 23.3 | 11 | 1 (9.1) | 12.4 | ---- | N/A | ---- | ---- | N/A | ---- | ---- | N/A | ---- |
| **No** | 424 | 3 (0.7) | ---- | 174 | 0 (0) | ---- | 43 | 0 (0) | ---- | ---- | N/A | ---- | ---- | N/A | ---- | ---- | N/A | ---- |

N/A, data not collected for controls.

Abbreviations: MCPP, microbiologically confirmed pneumococcal pneumonia; WB, whole blood; PCV, pneumococcal conjugate vaccine; CXR, chest radiograph; CXR-AC, alveolar consolidation on CXR; WBC, white blood cells; CRP, C-reactive protein; HIV, human immunodeficiency virus; PCR, polymerase chain reaction; NP, nasopharyngeal; OP, oropharyngeal; RTI, respiratory tract illness.

Restricted to case groups with WB pneumococcal PCR positive children.

^a^Non-confirmed cases defined as cases without isolation of bacteria from culture of blood, lung aspirate or pleural fluid, or PCR of lung aspirate or pleural fluid.

^b^ CXR+ defined as radiographic evidence of pneumonia (consolidation and/or other infiltrates).

^c^ Prior use of antibiotics defined as serum bioassay positive, antibiotic administration at the referral facility, or antibiotic administration prior to blood specimen collection at the study facility.

^d^ Hypoxemia was defined as <92% on room air (<90% at elevation, Zambia and South Africa) or a requirement for supplemental oxygen if a room air reading was not available.

**Supplementary Table 3:** Characteristics associated with whole blood pneumococcal PCR-positivity among general pediatric admissions to Kilifi County Hospital, all children

| **Characteristic** | **All Cases** | **Cases with pneumococcal bacteremia^a^** | **Cases without pneumococcal bacteremia^a^** | | |
| --- | --- | --- | --- | --- | --- |
|  |  |  | **With severe or very severe pneumonia^b^** | **Without severe or very severe pneumonia ^b,c^** | **Confirmed for another pathogen^d^** |
|  | **WB+ n/N (%)** | **WB+ n/N (%)** | **WB+ n/N (%)** | **WB+ n/N (%)** | **WB+ n/N (%)** |
|  |  |  |  |  |  |
| **Overall** | 274/6968 (3.9) | 37/60 (61.7) | 71/1694 (4.2) | 85/2905 (2.9) | 7/148 (4.7) |
|  |  |  |  |  |  |
| **Age** | **p=.003** | p=.29 | p=.11 | p=.27 | p=.94 |
| 0-28 d | 29/1379 (2.1) | 1/2 (50.0) | 8/337 (2.4) | 5/295 (1.7) | 1/40 (2.5) |
| 29 d < 6 m | 31/781 (4.0) | 2/2 (100.0) | 15/409 (3.7) | 6/179 (3.4) | 1/21 (4.8) |
| 6-11 m | 38/790 (4.8) | 5/9 (55.6) | 15/296 (5.1) | 11/337 (3.3) | 1/22 (4.6) |
| 12-23 m | 40/1075 (3.7) | 9/12 (75.0) | 11/257 (4.3) | 10/517 (1.9) | 1/24 (4.2) |
| 24-59 m | 68/1502 (4.5) | 10/22 (45.5) | 8/211 (3.8) | 20/727 (2.8) | 1/17 (5.9) |
| >60 m | 68/1441 (4.7) | 10/13 (76.9) | 14/184 (7.6) | 33/850 (3.9) | 2/24 (8.3) |
|  |  |  |  |  |  |
| **Pneumonia syndrome** | **p<.001** | **p=.017** | p=.98 | - | p=.74 |
| Severe | 56/972 (5.8) | 13/15 (86.7) | 35/833 (4.2) | - | 1/18 (5.6) |
| Very severe pneumonia syndrome | 68/1357 (5.0) | 14/21 (66.7) | 36/861 (4.2) | - | 1/39 (2.6) |
| Neither^c^ | 150/4635 (3.2) | 10/24 (41.7) | - | 85/2905 (2.9) | 5/90 (5.6) |
|  |  |  |  |  |  |
|  |  |  |  |  |  |
|  |  |  |  |  |  |
|  |  |  |  |  |  |
| **HIV-antibody positive** | **p<.001** | p=.78 | p=.18 | **p=.033** | p=.66 |
| Yes | 32/385 (8.3) | 9/15 (60.0) | 8/117 (6.8) | 9/158 (5.7) | 1/18 (5.6) |
| No | 217/5855 (3.7) | 25/39 (64.1) | 59/1402 (59) | 67/2437 (2.8) | 6/111 (5.4) |
|  |  |  |  |  |  |
| **NP culture or NP/OP PCR positive for Pneumococcus** | **p<.001** | p=.32 | **p=.021** | N/A | - |
| Yes | 51/886 (5.8) | 15/21 (71.4) | 30/627 (4.8) | N/A | 0/16 (0) |
| No | 5/364 (1.4) | 0/1 (0.0) | 4/259 (1.5) | N/A | 0/14 (0) |
|  |  |  |  |  |  |
| **Any virus co-infection (NP/OP)^e^** | p=.91 | p=.25 | p=.89 | N/A | - |
| Yes | 34/876 (3.9) | 8/14 (57.1) | 21/644 (3.3) | N/A | 0/15 (0) |
| No | 10/248 (4.0) | 4/4 (100.0) | 5/143 (3.5) | N/A | 0/10 (0) |
|  |  |  |  |  |  |
| **CXR+^f^** | **p<.001** | - | p=.054 | N/A | p=.364 |
| Yes | 12/60 (20.0) | 6/6 (100.0) | 5/46 (10.9) | N/A | 1/4 (25.0) |
| No | 11/276 (4.0) | 0/0 (0.0) | 9/226 (4.0) | N/A | 0/7 (0) |
|  |  |  |  |  |  |
| **Died in hospital** | p=.07 | p=.51 | p=.79 | p=.59 | p=.53 |
| Yes | 27/492 (5.5) | 8/11 (72.7) | 10/221 (4.5) | 4/143 (2.8) | 1/32 (3.1) |
| No | 247/6476 (3.8) | 29/49 (59.2) | 61/1473 (4.1) | 81/2762 (2.9) | 6/116 (5.2) |

Abbreviations: WB, pneumococcal whole blood; CXR+, chest radiograph positive; NP, nasopharyngeal; NP/OP, nasopharyngeal/oropharyngeal;

^a^ Pneumococcus isolated on blood culture specimen.

^b^ Children who did not have cerebrospinal fluid collected. No pathogens were identified on blood culture.

^c^ Not severe or very severe pneumonia syndrome includes children with any other medical cause of admission, including other respiratory illnesses.

^d^ Confirmed for another pathogen by culture of blood or cerebrospinal fluid.

^e^ Respiratory viruses tested by multiplex PCR include respiratory syncytial virus A and B; parainfluenza viruses 1, 2, 3, and 4; coronaviruses OC43, NL63, 229E and HKU; human metapneumovirus A and B; human bocavirus; influenza viruses A, B and C; parecho/enterovirus; cytomegalovirus; and adenovirus.

^f^ CXR results were included where children with WHO severe or very severe pneumonia had had a digitalised CXR and standardised reporting according to WHO methods was available. A greater number of CXR results became available after March 2012 when a mobile CXR unit was procured.

P-values obtained by chi-square or Fisher’s exact test for difference in whole blood pneumococcal PCR positivity by characteristic within each sub-group. Bold indicates p<.05.

N/A indicates data not systematically collected for the sub-group.

**Supplementary Figure 1** Flow diagram of enrolment for general paediatric admissions to Kilifi County Hospital, December 2010 to December 2013


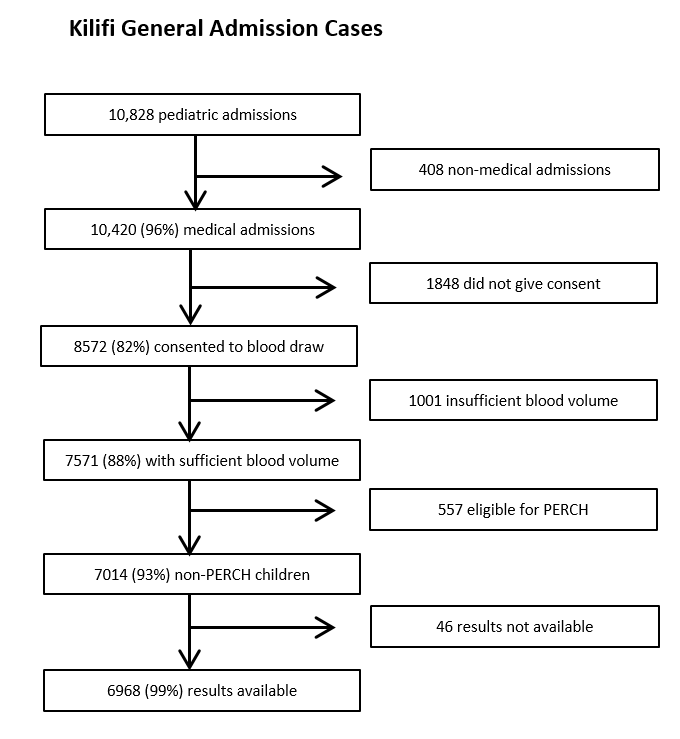


**Supplementary Figure 2a.** Proportion of PERCH whole blood specimens positive for pneumococcus by PCR by specimen collection date
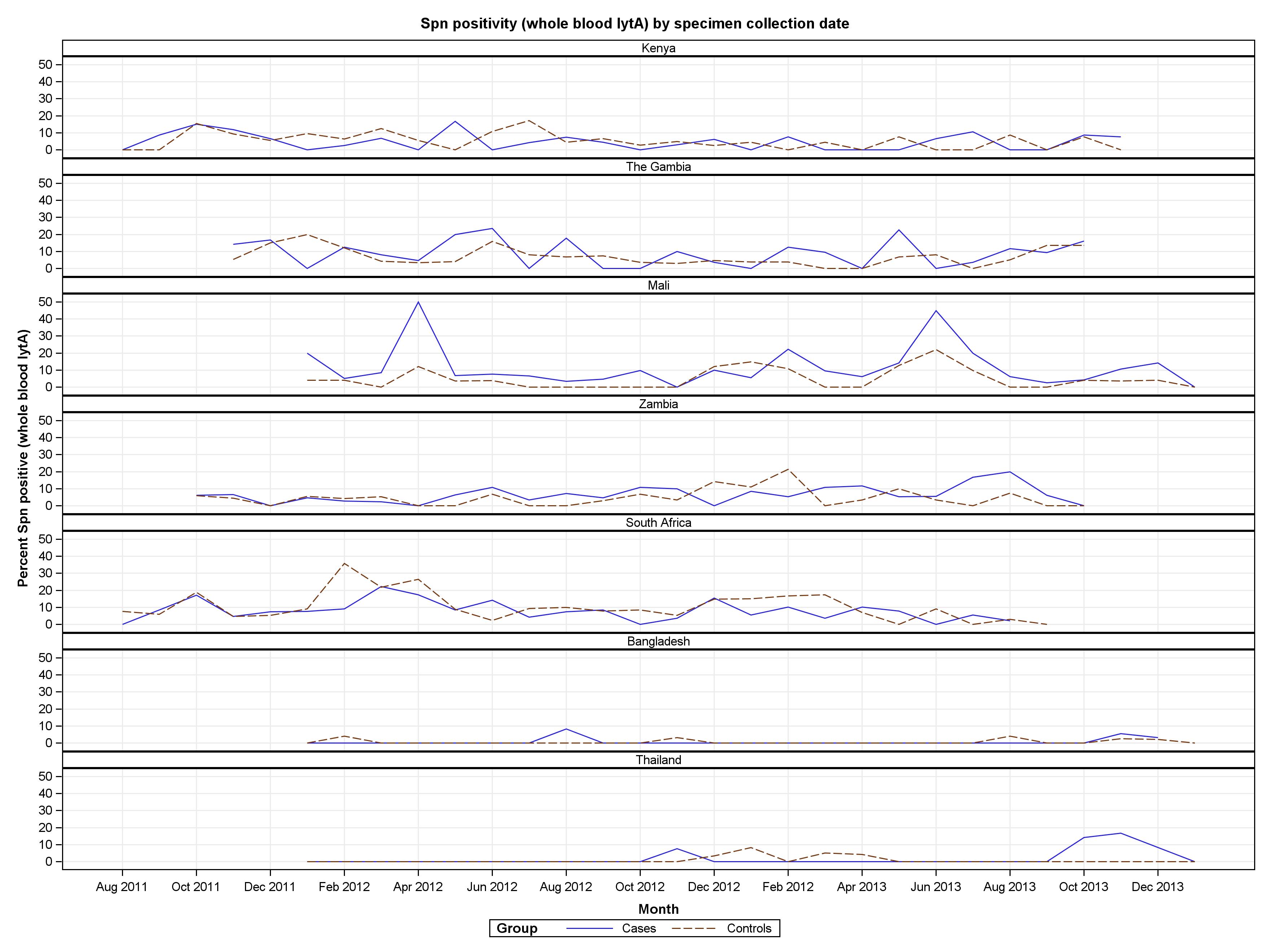


Lines correspond to enrollment period at the site.

Abbreviations: PCR, polymerase chain reaction; Spn, pneumococcus.

**Supplementary Figure 2b.** Proportion of PERCH whole blood specimens positive for pneumococcus by PCR by nucleic acid extraction date


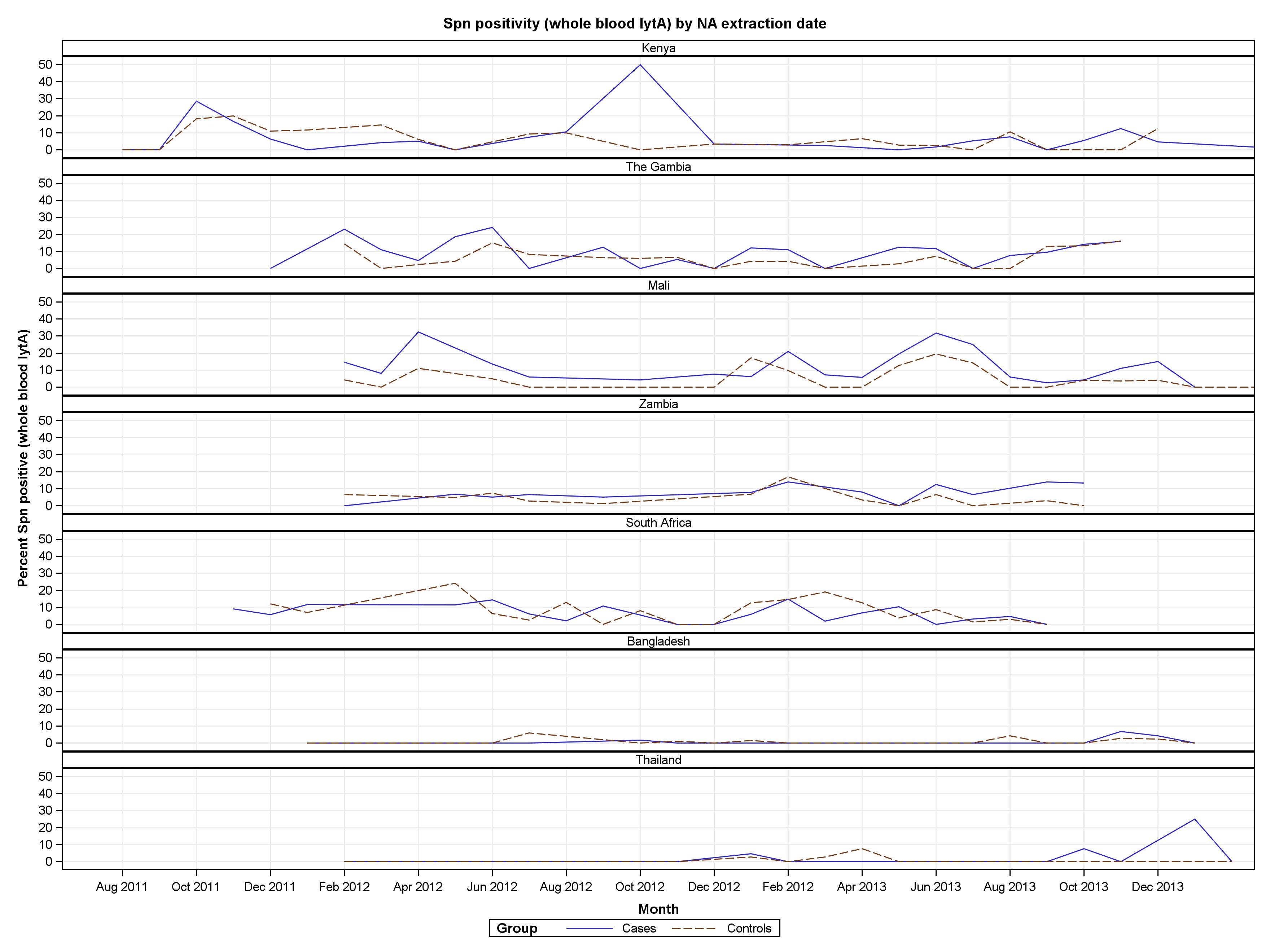


Lines correspond to enrollment period at the site.

Abbreviations: PCR, polymerase chain reaction; Spn, pneumococcus.

**Supplementary Figure 2c.** Proportion of PERCH whole blood specimens positive for pneumococcus by PCR by PCR run date


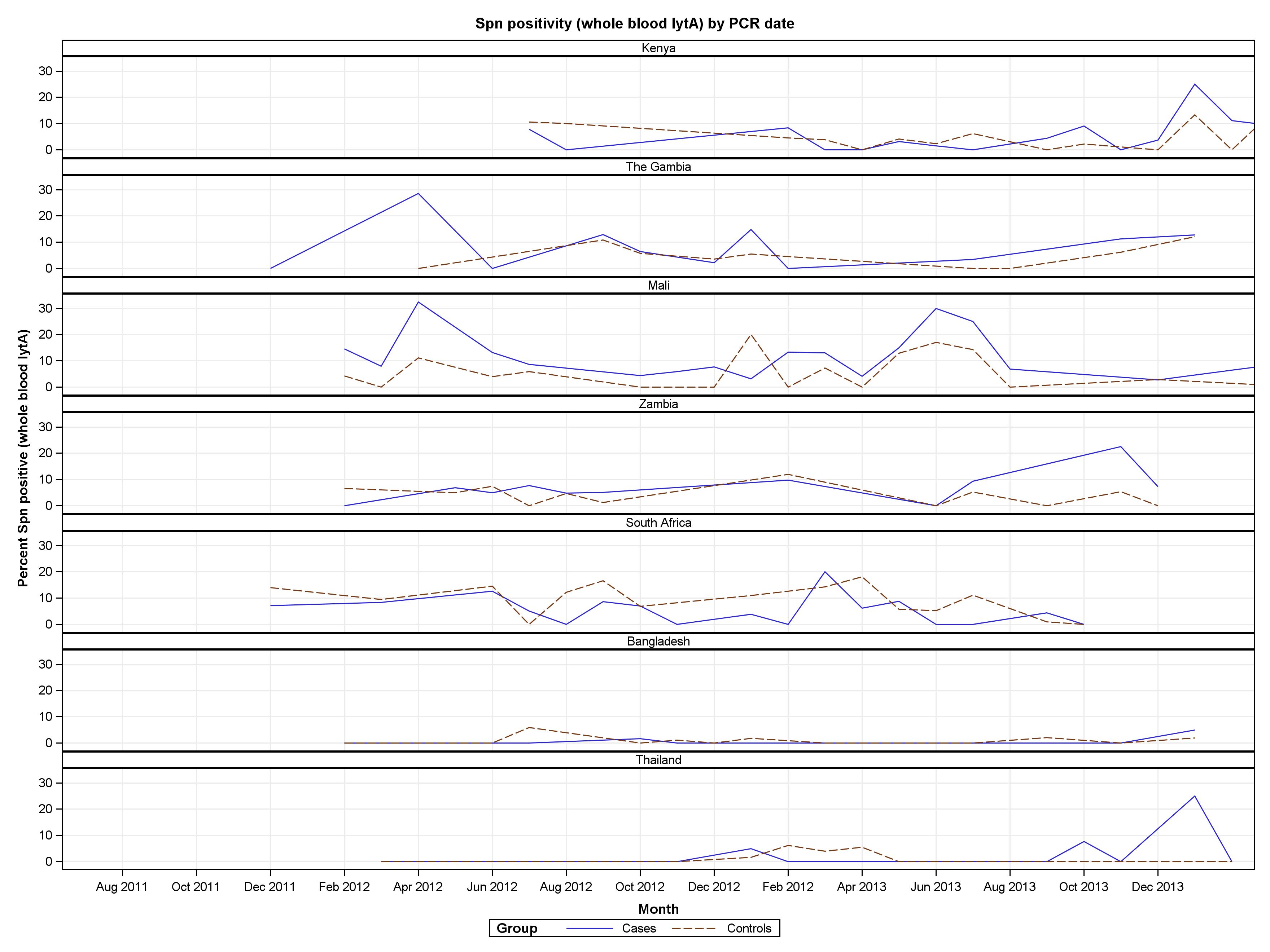


Lines correspond to enrollment period at the site. Abbreviations: PCR, polymerase chain reaction; Spn, pneumococcus.

**Acknowledgements:**

***PERCH Expert Group.*** William C. Blackwelder, Harry Campbell, John A. Crump, Adegoke Falade, Menno D de Jong, Claudio Lanata, Kim Mulholland, Shamim Qazi, Cynthia G. Whitney.

***Pneumonia Methods Working Group.*** Robert E Black, Zulfiqar A Bhutta, Harry Campbell, Thomas Cherian, Derrick W Crook, Menno D de Jong, Scott F Dowell, Stephen M Graham, Keith P Klugman, Claudio F Lanata, Shabir A Madhi, Paul Martin, James P Nataro, Franco M Piazza, Shamim A Qazi, and Heather J Zar.

***PERCH Chest Radiograph Reading Panel***

**Readers:** Dr. Kamrun Nahar, Dr. Fariha Bushra Matin, Dr. Claire Oluwalana, Dr. Bernard Ebruke, Dr. Joyce Sande, Dr. Micah Silaba Ominde, Dr. Mahamadou Diallo, Dr. Breanna Barger-Kamate, Dr. Nasreen Mahomed, Dr. David P. Moore, Dr. Anchalee Kruatrachue, Dr. Piyarat Suntarattiwong, Dr. Musaku Mwenechanya, Dr. Rasa Izadnegahdar, **Arbitrators:** Dr. Vera Manduku, Dr. John DeCampo, Dr. Marg DeCampo, Dr. Fergus Gleeson.

***PERCH Contributors:***

**Bangladesh:** Kamrun Nahar, Arif Uddin Sikdir, Sharifa Yeasmin, Dilruba Ahmed, Muhammad Ziaur Rahman, Muhammad Yunus, Muhammad Al Fazl Khan, Muhammad Jubayer Chisti, Abu Sadat Muhammad Sayeem, Shahriar Bin Elahi, Mustafizur Rahman; **The Gambia:** Michel Dione, Emmanuel Olutunde, Peter Githua, Ogochukwu Ofordile, Rasheed Salaudeen, David Parker; **Kenya:** Shebe Mohamed, Siti Ndaa, Micah Silaba, Neema Muturi, Angela Karani, Sammy Nyongesa, Anne Bett, Daisy Mugo, Salim Mwarumba, Robert Musyimi, Andrew Brent, James Nokes, David Mulewa, Joyce Sande, John Odhiambo, Joshua Wambua, Nuru Kibirige, Caroline Mulunda, Hellen Mjalla, Norbert Katira, Karen Dama, Loice Masha, Christine Mutunga, Mwanajuma Ngama, Stephen Mangi, Riziki Anthony, Mwarua Yubu, Elijah Wakili, Benson Katana, Shoboi Mgunya, Emmanuel Mumba, Benedict Mver, George Kuria, Felix Githinji, Norbert Kihuha, Boniface Jibendi, Tahreni Bwanaali, Agustus Kea; **Mali:** Nana Kourouma, Aliou Toure, Mahamadou Diallo, Breana Barger-Kamate, Mariam Samake, Seydou Sissoko, Abdoul Aziz Maiga, Mariam Samake, Toumani Sidibe, Mariam Sylla, Aziz Diakite, Bassirou Diarra; **South Africa:** Azwidihwi Takalani, Andrea Hugo, Susan Nzenze, Ndulela Titi, Mmabatho Selela, Malebo Motiane, Minah Nkuna, Nonhlanhla Tsholetsane, Sibonsile Moya, Debra Katisi, Tondani Netshishivhe, Lerato Mapetla, Gudani Singo, Simphiwe Gasa, Cece Mgenge, Nozipho Mthunzi, Nombulelo Monedi, Tanja Adams, Shafeeka Mangera, Jeannette Wadula, Peter Tsaagane, Jenifer L. Vaughan, Sakina Loonat, Martin Hale, Sugeshnee Pather, Mariëtte Middel, Siobhan Trenor, Palesa Morailane, Ntombi Maya, Rene Sterley, Charné Combrinck, Given Malete, Lerato Qoza, Grizelda Liebenberg, Hendrik van Jaarsveld, Zunaid Kraft, Lisa-Marie Mollentze, Lourens Combrinck, Tsholofelo Mosome; **Thailand:** Sununta Henchaichon, Dr. Tussanee Amornintapichet, Dr. Somchai Chuananont, Toni Whistler, Juraiporn Ratanodom, Patranuch Sapchookul, Ornuma Sangwichian, Sirirat Makprasert, Manoon Hirunsalee, Possawat Jorakate, Anek Kaewpan, Duangkamol Siludjai, Apiwat Lapamnouysup, Dr. Wantana Paveenkittiporn, Waraporn Ubonphen, Dr. Peera Areerat, Ms.Yupapan Wannachaiwong, Ms Tewa Faipet, Ms Punnat Natnarakorn, Ms Ahchanan Sacharone, Mr.Winai Makmool, Ms. Kanlaya Sornwong, Ms. Promporn Sansuriwong, Ms. Ratchanida Potiya, Ms. Wasana Hongsawong, Ms.Wipa Matchaikhen, Ms. Thatsanawan Chaiyabil, Ms.Piyapai Wannarach, Ms Chamaiporn Wadeesirisak, Mr. Yuttapong Norapet, Mattana Bangkung, Mr. Barameht Piralam, Sathapana Naorat, Anchalee Jatapai, Prasong Srisaengchai, Dr. Leonard Peruski, Ms.Dawan Phaensoongnoen, Ms.Tussaaorn Klangprapan, Ms.Narawadee Dumrongdee, Ms.Atchara Srithongkham, Mr. Piyawut Noinont, Ms. Pornthip Kamlee, Ms.Siyapa Mongkornsuk; **Zambia:** Justin Mulindwa, Musaku Mwenechanya, John Mwaba, Magdalene Mwale, Julie Duncan, Kazungu Siazele, Muntanga Mapeni, Emily Hammond; **Canterbury Health Laboratory, Christchurch, New Zealand:** Rose Watt, Shalika Jayawardena; **The Emmes Corporation, Rockville, Maryland:** Mark Wolff, Megan Sanza, Omid Neyzari.
